# Safety of biological and targeted synthetic disease-modifying antirheumatic drugs for rheumatoid arthritis as used in clinical practice: results from the ARTIS programme

**DOI:** 10.1136/ard-2022-223762

**Published:** 2023-02-14

**Authors:** Thomas Frisell, Hannah Bower, Matilda Morin, Eva Baecklund, Daniela Di Giuseppe, Benedicte Delcoigne, Nils Feltelius, Helena Forsblad-d'Elia, Elisabet Lindqvist, Ulf Lindström, Johan Askling, the ARTIS Study group, Gerd-Marie Ahlenius

**Affiliations:** 1 Clinical Epidemiology Division, Department of Medicine Solna, Karolinska Institutet, Stockholm, Sweden; 2 Department of Medical Sciences, Uppsala University, Section of Rheumatology, Uppsala, Sweden; 3 Department of Public Health and Caring Sciences, Uppsala University, Uppsala, Sweden; 4 Department of Rheumatology and Inflammation Research, Institute of Medicine, Sahlgrenska Academy, University of Gothenburg, Gothenburg, Sweden; 5 Section of Rheumatology, Department of Clinical Sciences Lund, Lund University, Skane University Hospital, Lund University, Lund, Sweden; 6 Rheumatology, Theme Inflammation and Ageing, Karolinska University Hospital, Stockholm, Sweden

**Keywords:** Antirheumatic Agents, Biological Therapy, Arthritis, Rheumatoid, Cardiovascular Diseases, Epidemiology

## Abstract

**Objective:**

Longitudinal clinical registry-infrastructures such as Anti-Rheumatic Therapies in Sweden (ARTIS) allow simultaneous comparison of the safety of individual immunomodulatory drugs used in clinical practice, with consistent definitions of treatment cohorts, follow-up and outcomes. Our objective was to assess and compare incidence rates of key safety outcomes for individual targeted synthetic or biological disease-modifying antirheumatic drugs (b/ts DMARDs) in rheumatoid arthritis (RA), updating previous reports and including newer treatments including Janus Kinase inhibitors (JAKi).

**Methods:**

Nationwide register-based cohort study including all patients with RA in Sweden registered as starting any b/tsDMARD 1 January 2010 through 31 December 2020, followed until 30 June 2021 (N=20 117). The incidence rates of selected outcomes, identified through national healthcare registers, were compared between individual b/tsDMARDs, adjusted for confounding by demographics, RA disease characteristics and comorbidity.

**Results:**

There were marked differences in treatment discontinuations due to adverse events (rates per 1000 person-years ranged from 18 on rituximab to 57 on tofacitinib), but few significant differences were observed for the serious adverse events under study. Neither cardiovascular events nor general serious infections were more frequent on baricitinib or tofacitinib versus bDMARDs, but JAKi were associated with higher rates of hospital-treated herpes zoster (HR vs etanercept, 3.82 (95% CI 2.05 to 7.09) and 4.00 (1.59 to 10.06)). Low number of events limited some comparisons, in particular for sarilumab and tofacitinib.

**Conclusion:**

Data from ARTIS supports that the b/tsDMARDs currently used to treat RA have acceptable and largely similar safety profiles, but differences exist in particular concerning tolerability and specific infection risks.

**WHAT IS ALREADY KNOWN ON THIS TOPIC**

- By enabling structured follow-up of large cohorts of patients representative of those treated in clinical practice, postapproval analyses of real-world data play a critical role in the evaluation of the safety, and of the relative safety, of antirheumatic drugs.
- Anti-Rheumatic Therapies in Sweden (ARTIS) is a long-standing register-based drug evaluation framework, enabling the simultaneous comparison of the safety profiles of individual targeted synthetic or biological disease-modifying antirheumatic drug (b/tsDMARD) used in clinical practice against rheumatoid arthritis (RA), with consistent cohort definitions, follow-up and data capture across drugs.

**WHAT THIS STUDY ADDS**

- We present incidence rates and relative risks of 10 key safety outcomes for individual b/tsDMARDs used to treat RA over the last decade, updating previous reports and extending analyses to newer treatments.

**HOW THIS STUDY MIGHT AFFECT RESEARCH, PRACTICE OR POLICY**

- Our results support that the currently available b/tsDMARDs have acceptable and on the whole similar safety profiles in a real-world population, with some differences concerning tolerability, specific infection risks and certain serious but rare outcomes.
- ARTIS and similar register-based safety monitoring programmes can provide comparative safety data across all treatment options used in clinical practice, which is instrumental for the postmarketing safety evaluation of recent as well as established immunomodulatory drugs in rheumatology.

## Introduction

Over a dozen approved targeted synthetic or biological disease-modifying antirheumatic drugs (b/tsDMARDs) are available for the treatment of rheumatoid arthritis (RA).^1^ The choice between these drugs should ideally be based on the risk-benefit balance of each drug versus the others for the individual patient.

In practice, the evidence that informs this choice is, even on a population level, limited.^1–3^ While superior/non-inferior efficacy of one treatment over another can be demonstrated in relatively small studies with limited follow-up, many safety concerns require larger studies and longer follow-up times for differences to become clear, even when the induction time for a given safety event itself is not an issue. This is recognised by the regulatory framework where data from pivotal randomised controlled trials (RCTs) are usually considered sufficient for demonstrating the efficacy and non-toxicity of the drug, but postapproval safety studies (PASS) are required for several years to evaluate drug-associated risks.^4^

Much of what we currently know about risks associated with individual b/tsDMARDs thus come from observational studies comparing rates of adverse events among patients treated with different drugs in clinical practice.^2^ Since—in clinical practice—the choice is neither between one drug versus all others nor between one class versus another class, but always between all individual available treatment options, it is unfortunate that many research studies, and certainly the vast majority of all PASS studies, are designed to compare a single drug to all other drugs grouped together or limited to just one or two specific alternative options.^5^ Comparison of results across studies are then necessary to draw conclusions about the available options’ relative safety vs each other. Such between-study comparisons and extrapolations are, however, inherently difficult since both target populations and outcome rates may differ substantially by cohort inclusion and exclusion criteria, variable definitions, method of data capture and also by analytical approach.^6^ Therefore, whereas analytic methods may effectively accommodate confounding by indication *within* a study, it is far from evident that such methods guarantee comparability *across* studies.

Long-standing drug registers covering all individual treatment options for a disease, such as the Anti-Rheumatic Therapies in Sweden (ARTIS),^7^ allowing the simultaneous comparison of each drug available for use in clinical practice, with consistent definitions of treatment cohorts, follow-up and outcome capture, have a critical role in the evaluation of the relative safety of b/tsDMARDs to inform individual risk-benefit assessment.

To enable the clinically much-needed direct comparisons across all available b/tsDMARDs approved for RA, we therefore investigated absolute and relative rates of key safety outcomes for all individual b/tsDMARDs available for the treatment of RA.

## Methods

This nationwide register-based cohort study included all patients with RA in Sweden who were recorded as starting any b/tsDMARD between 1 January 2010 and 31 December 2020, and followed them until 30 June 2021 to compare the incidence of selected outcomes between individual treatments while adjusting for a range of potential confounders.

### Data sources

The ARTIS safety monitoring programme is described in online supplemental file 1 and is constructed by linking individual-level longitudinal data on treatments, disease activity and other clinical measurements from the Swedish Rheumatology Quality Register (SRQ),^7^ covering around 90% of all b/tsDMARD initiations in Sweden,^8^ to prospectively collected data in Swedish national healthcare registers.^9^ This includes data on diagnoses recorded in inpatient and outpatient specialist care from the National Patient Register, all filled prescriptions in community pharmacies from the Prescribed Drug Register and demographics and migration dates from census/taxation registers.

10.1136/ard-2022-223762.supp1 Supplementary data

### Treatment exposure

All approved b/tsDMARDs used for RA in Sweden during the study period were included: antitumour necrosis factor (TNFi) bDMARDs: adalimumab, certolizumab pegol, etanercept, golimumab and infliximab; other bDMARDS: abatacept, anakinra, rituximab, sarilumab, tocilizumab and the Janus Kinase inhibitors (JAKi) tsDMARDs: baricitinib, tofacitinib and upadacitinib. Drugs with fewer than 200 treatment episodes (here: anakinra (n=84) and upadacitinib (n=105)) were excluded from further analysis. Patients were considered exposed to a treatment from their first ever start of that specific b/tsDMARD, as recorded in the SRQ, until treatment switch or discontinuation. When a patient switched or discontinued treatment, we added a lag time of 90 days after the treatment was stopped (183 days for rituximab) to capture adverse events linked to treatment discontinuation but registered with some delay. Treatment stop date was defined as the first of: recorded stop in the SRQ, recorded start of another b/tsDMARD in the SRQ and filled prescription of another b/tsDMARD in the Prescribed Drug Register. If restarted within 90 days (183 days for rituximab), the two treatment episodes were merged. We did not differentiate between biosimilar versions of each drug, and switches between such were not considered treatment discontinuations. Patients could contribute with multiple treatment episodes on different drugs, but only the first ever start for each molecule.

Follow-up was censored at death or first emigration from Sweden after treatment start.

### Comparator cohorts

A general population comparator group was drawn 1:5 age-sex-region matched to combined b/tsDMARD-treated cohort and free of chronic inflammatory joint disease at the index persons’ date of treatment start. General population comparator subjects inherited the start date from their matched index individual with RA and were censored at death, emigration or any first recorded diagnosis of RA.

A cohort of b/tsDMARD-naïve patients with RA was defined as all patients with at least two separate dates of diagnosis with RA in the National Patient Register, with start date being the earliest of their second diagnosis date and 1 January 2010 and censored at the first ever recorded start of a b/tsDMARD. This cohort lacked data on disease activity.

### Outcomes

Ten study outcomes were defined to capture important known or suspected risks associated with b/tsDMARD treatment: (1) treatment discontinuation due to adverse events, (2) major adverse cardiovascular events (MACE, including acute coronary syndrome (ACS), stroke and fatal cardiovascular events), (3) serious (requiring inpatient treatment) infection, (4) herpes zoster registered in specialty care, (5) tuberculosis, (6) non-steatosis liver disease, (7) diagnosed depression, (8) attempted or completed suicide, (9) any hospitalisation and (10) all-cause mortality. Reason for treatment discontinuation was recorded in the SRQ, laboratory-confirmed tuberculosis was retrieved from the Swedish Public Health Agency’s register of communicable diseases; all other outcomes were defined by recorded diagnosis in the National Patient Register, covering inpatient and specialist outpatient care but not general practitioners, or as cause of death (definitions in online supplemental table 1). Malignancies and thromboembolic events were omitted as they have been the subject of recent publications from ARTIS.^10 11^

Patients with a recent history of an outcome (prior 5 years, except for infection where only last year was considered) were excluded from analyses of the same outcome, except in analysis of discontinuation due to adverse events.

### Covariates

Covariates were selected to broadly capture demographics, comorbidity and RA-related characteristics including disease activity. Census registries provided data on age, sex, immigration status and highest achieved education. SRQ provided data on smoking, RF/anti-citrullinated peptide antibodies (ACPA), RA duration, previous b/tsDMARD use, comedication with conventional synthetic DMARDs and glucocorticoids, the 28-joint disease activity score (DAS28-CRP) and the Health Assessment Questionnaire-Disability Index (HAQ). Comorbidity or medical history was assessed during the 5 years up until treatment start by registrations of ICD-10 diagnosis codes for malignancy, infections, joint surgery, chronic pulmonary disease, diabetes, cardiovascular disease, depression and the sum of prior days hospitalised. Different lookback period was sued for serious infections (1 year), joint surgery and malignancy (10 years). Detailed definitions are given in online supplemental table 2.

### Statistical analyses

Crude and adjusted incidence rates were calculated for all outcomes and treatment groups. Only the first event in each treatment episode was counted. Cox regression by time since treatment start was used to estimate the HR using the largest treatment cohort (etanercept) as reference. HRs are only presented for contrasts with more than five observed events in both groups.

Incidence rates and Cox regressions were adjusted with stabilised inverse probability of treatment weights constructed as the inverse of the predicted probability to have received the treatment actually received, multiplied by the sample proportion with the same treatment.^12^ Weights were truncated to the 99th percentile. Probabilities were predicted by multinomial logistic regression, regressing all covariates on treatment cohort. Balance was checked preweighting and postweighting (population standardised difference <0.1 was considered good balance). To allow comparison, standard multivariable Cox regression were run with the same variables and parameterisations.

Data were complete on treatments, outcomes and most covariates derived from national registers, but about 30% lacked data on baseline DAS28 and HAQ. Missing covariate data were accounted for by multiple imputation through chained equations, using fully conditional specifications with logistic models for categorical variables and predicted mean matching for continuous. We imputed 20 data sets, with 10 burn-in iterations. All covariates were parameterised as in the weight models, with second degree polynomials for continuous variables, and included the treatment assignment and all outcomes (event indicator and Nelson-Aalen estimate of the cumulative hazard). Robust SEs were used to calculate 95% CIs for all HRs, thus correcting for the weighting and the potential inclusion of the same patient in multiple treatment group, and combined across imputed dataset using Rubin’s rule. Analyses were performed in SAS V.9.4 (SAS Institute, Cary, North Carolina, USA).

### Sensitivity analysis

The impact of the study period was tested by restriction to: (1) the time after JAKi market entry (excluding all b/tsDMARD starts before 1 January 2017) and (2) the time before the COVID-19 pandemic (follow-up terminated at 28 February 2020).

### Patient and public involvement

Patient representatives were not involved in the design or interpretation of this study.

## Results

Over the 11-year study inclusion period, 2010–2020, we included 20 117 unique patients with RA who started at least one b/tsDMARD, contributing a total of 34 279 treatment episodes. The most commonly initiated b/tsDMARDs were the TNFi etanercept and adalimumab, while the recently introduced anti-IL-6R sarilumab was the least commonly started among the included treatments (table 1). Due to differences in market entry, the average follow-up per patient was about 3 years for most bDMARDs, below 2 years for JAKi and lowest for sarilumab, 1.3 years.

**Table 1:** Patient characteristics at start of b/tsDMARD therapy, among all Swedish patients with RA, 2010–2020

	Etanercept	Adalimumab	Infliximab	Certolizumab	Golimumab	Abatacept	Rituximab	Tocilizumab	Sarilumab	Baricitinib	Tofacitinib
N patients	8748	5526	2971	2179	1889	3434	4220	2757	292	1837	426
Person-years of exposure	26 508.3	13 566.1	8217.7	6180.3	6051.1	8799.8	15 821.5	8089.8	380.5	3401.8	648.9
Mean (SD)	3.0 (2.8)	2.5 (2.7)	2.8 (2.9)	2.8 (3.0)	3.2 (3.1)	2.6 (2.5)	3.7 (3.1)	2.9 (3.0)	1.3 (0.9)	1.9 (1.3)	1.5 (1.3)
Demographics											
Age, mean (SD)	58 (14)	58 (14)	58 (14)	56 (15)	57 (14)	61 (13)	64 (13)	59 (14)	59 (14)	61 (14)	59 (13)
Female, %	77	76	74	78	78	80	75	79	79	82	82
Highest education, %											
9 years or less	9	8	13	9	10	11	14	11	8	9	5
10 years to 12 years	58	58	60	57	58	59	59	59	64	58	65
>12 years	33	34	27	33	32	29	27	30	28	33	30
Swedish-born, %	86	86	82	88	86	86	84	86	84	85	87
Ever smoker, %	58	58	60	57	54	61	64	58	58	59	59
RA clinical characteristics											
Rheumatoid factor positive, %	72	71	73	73	73	76	86	76	74	74	71
Disease duration, years, mean (SD)	7.7 (10.6)	8.4 (11.5)	6.8 (10.8)	8.3 (12.1)	8.9 (10.9)	11.7 (11.7)	12.7 (11.7)	10.5 (11.0)	11.0 (10.2)	13.2 (11.7)	13.1 (11.1)
DAS28, mean (SD)	4.3 (1.2)	4.2 (1.2)	4.5 (1.2)	4.4 (1.2)	4.3 (1.3)	4.5 (1.2)	4.5 (1.2)	4.8 (1.2)	4.5 (1.2)	4.3 (1.1)	4.5 (1.3)
HAQ, mean (SD)	1.0 (0.6)	1.0 (0.6)	1.0 (0.7)	1.0 (0.6)	1.0 (0.7)	1.3 (0.6)	1.3 (0.7)	1.3 (0.6)	1.3 (0.6)	1.1 (0.7)	1.3 (0.7)
Conc. MTX, %	59	60	76	56	66	52	53	48	45	39	35
Conc. non-MTX csDMARD, %	16	14	18	15	15	13	17	11	9	10	7
Conc. oral steroids, %	43	40	44	47	41	52	54	53	38	45	52
Prior b/tsDMARDs, %											
0	62	50	75	53	48	18	31	16	6	15	9
1–2	32	42	20	33	39	51	46	53	50	38	29
3+	6	8	5	15	13	31	22	31	44	47	63
Medical history*											
Malignancy, %	3.6	3	3.1	3.5	2.3	5	11.1	4	2.4	4.4	4.5
Serious infection, %	9.2	8.7	8.5	9.3	9.1	18.2	17.3	12.2	11.3	15.1	14.8
Serious herpes zoster %	1	0.7	0.5	0.8	1	1	1.2	0.9	0.7	1	0.9
Joint surgery, %	8.6	8.4	8.3	9.5	9.1	13	13.6	12.7	13	12.8	14.8
COPD, %	2.8	2.2	2.6	2.2	1.7	5.4	5.6	3.4	3.1	4.2	4
Diabetes mellitus, %	7.2	7.1	6	6.7	5.7	10.4	9.4	7.7	9.6	8	8.9
ACS, %	1.7	1.8	1.6	1.3	1.7	3	2.8	2.2	2.7	2.6	1.6
Stroke, %	1	1	1	1.1	1	1.5	2.1	1.3	0.3	1.3	0.9
Days hospitalised, %											
0	75	76	74	72	73	61	57	66	75	67	64
1–9	16	15	17	18	17	21	22	19	15	19	23
10+	9	8	9	10	10	18	22	15	10	14	13

*Medical history in 5 years before treatment start, except serious infection (1 year before start) and malignancy or joint surgery (10 years before).

ACS, acute coronary syndrome; COPD, chronic obstructive lung disease; csDMARD, conventional synthetic disease-modifying antirheumatic drug; HAQ, Health Assessment Questionnaire-Disability Index; RA, rheumatoid arthritis.

### Evidence for channelling to therapy

In keeping with past and current treatment guidelines, TNFi were more often used as a first line b/tsDMARD (in particular infliximab), while sarilumab and the JAKi were predominately used later in the treatment course (table 1). TNFi initiators also had lower disease activity, less comorbidity and more concomitant conventional synthetic DMARD use compared with initiators of other modes of action. Rituximab was more common among older and RF/ACPA-positive patients. Rituximab initiators also had the highest comorbidity burden, followed by abatacept initiators who had similar rates of non-malignancy comorbidity.

### Incidence rate by b/tsDMARD

**Figure 1:**
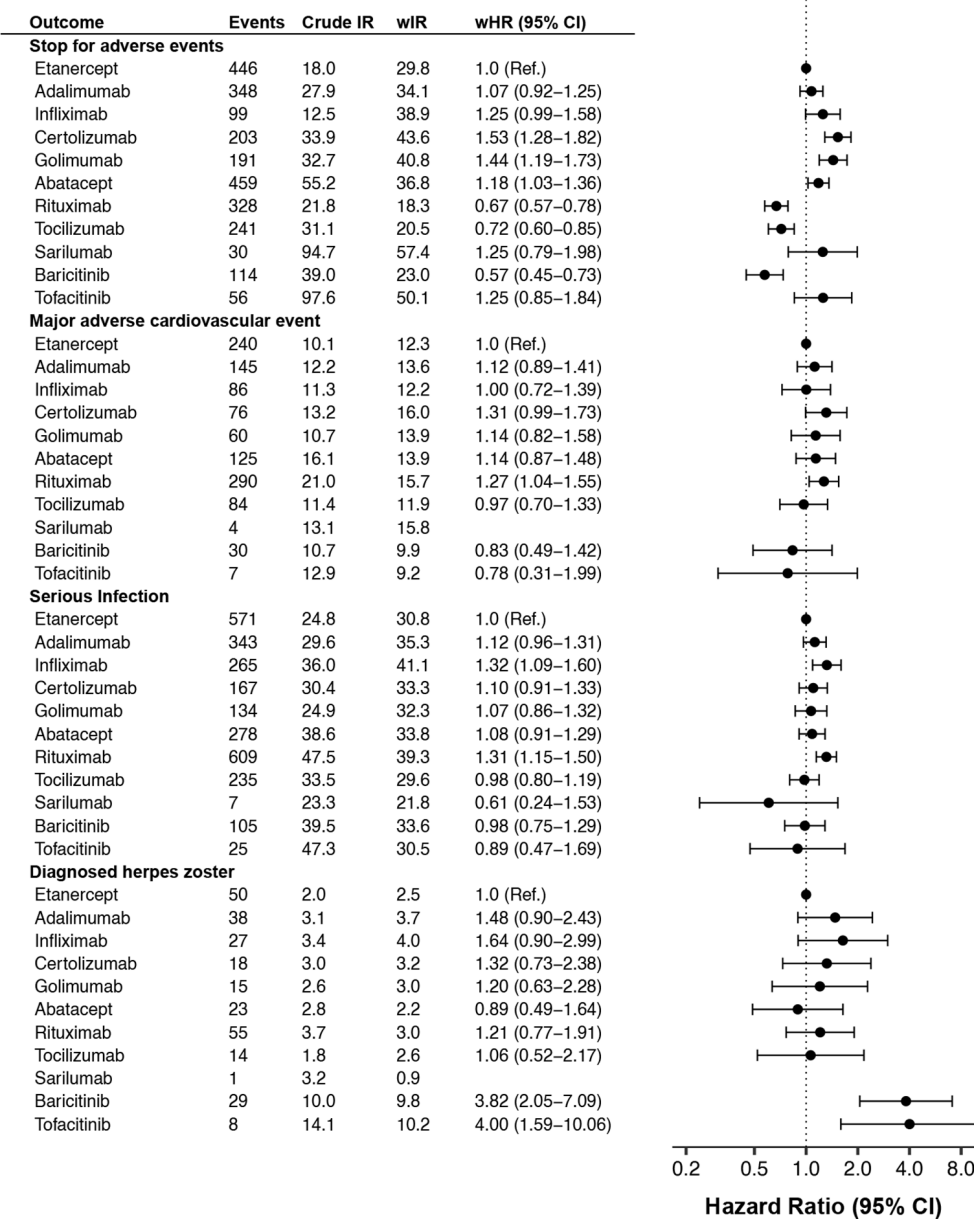
Crude and weighted incidence rate per 1000 person-years of selected safety outcomes by b/tsDMARD, and adjusted HRs versus etanercept, among all Swedish patients with RA who started treatment 2010–2020, followed until 30 June 2021. b/tsDMARDs, targeted synthetic or biological disease-modifying antirheumatic drugs; wHR, weighted HR from Cox regression; wIR, inverse probability of treatment weighted incidence rate per 1000 person-years, adjusted for demographics, RA clinical characteristics and comorbidity; RA, rheumatoid arthritis.

**Figure 2:**
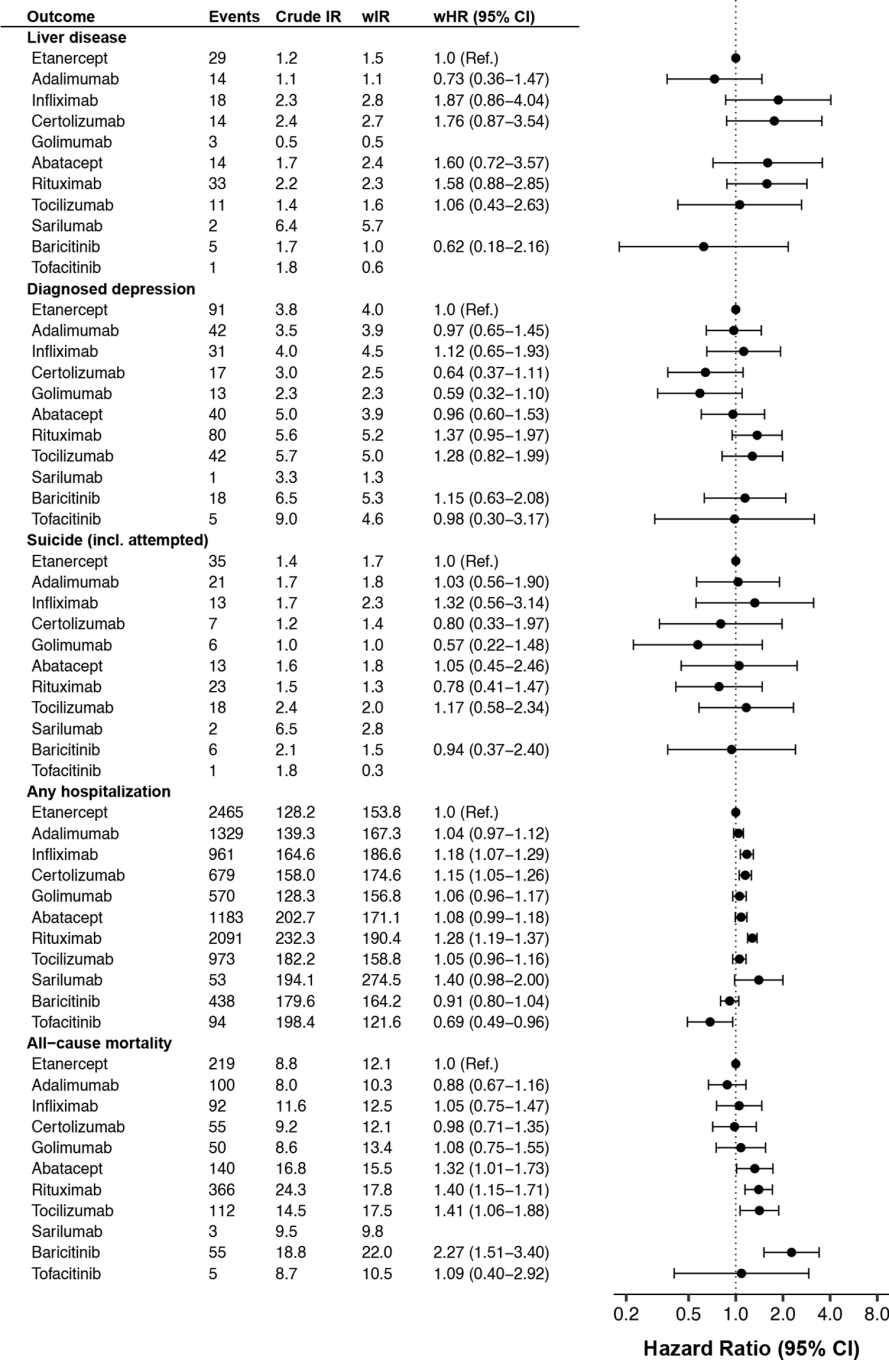
Crude and weighted incidence rate per 1000 person-years of selected safety outcomes by b/tsDMARD, and adjusted HRs vs etanercept, among all Swedish patients with RA who started treatment 2010–2020, followed until 30 June 2021. b/tsDMARDs, targeted synthetic or biological disease-modifying antirheumatic drugs; wHR, weighted HR from Cox regression; wIR, inverse probability of treatment weighted incidence rate per 1000 person-years, adjusted for demographics, RA clinical characteristics and comorbidity; RA, rheumatoid arthritis.

As expected, channelling resulted in large differences in the crude incidence of several outcomes. For instance, the crude rates of several age-related outcomes (including all-cause mortality, serious infections, and MACE) were about twice as high on rituximab than on etanercept (figures 1 and 2, crude HRs in online supplemental table 5). These observed risk differences were largely explained by differences in patient characteristics, as evident from the much greater inter-drug similarity in weighted incidence rates.

After adjusting for baseline patient characteristics, substantial differences remained in the rate of treatment discontinuation due to adverse events (figure 1). The weighted incidence rate was 30 per 1000 person-years (PYs) on etanercept (ie, the predicted rate had the whole b/tsDMARD-treated RA population used etanercept), but the corresponding rate was 18%–53% higher on abatacept, infliximab, golimumab and certolizumab pegol and 28%–43% lower on tocilizumab, rituximab and baricitinib.

**Figure 3:**
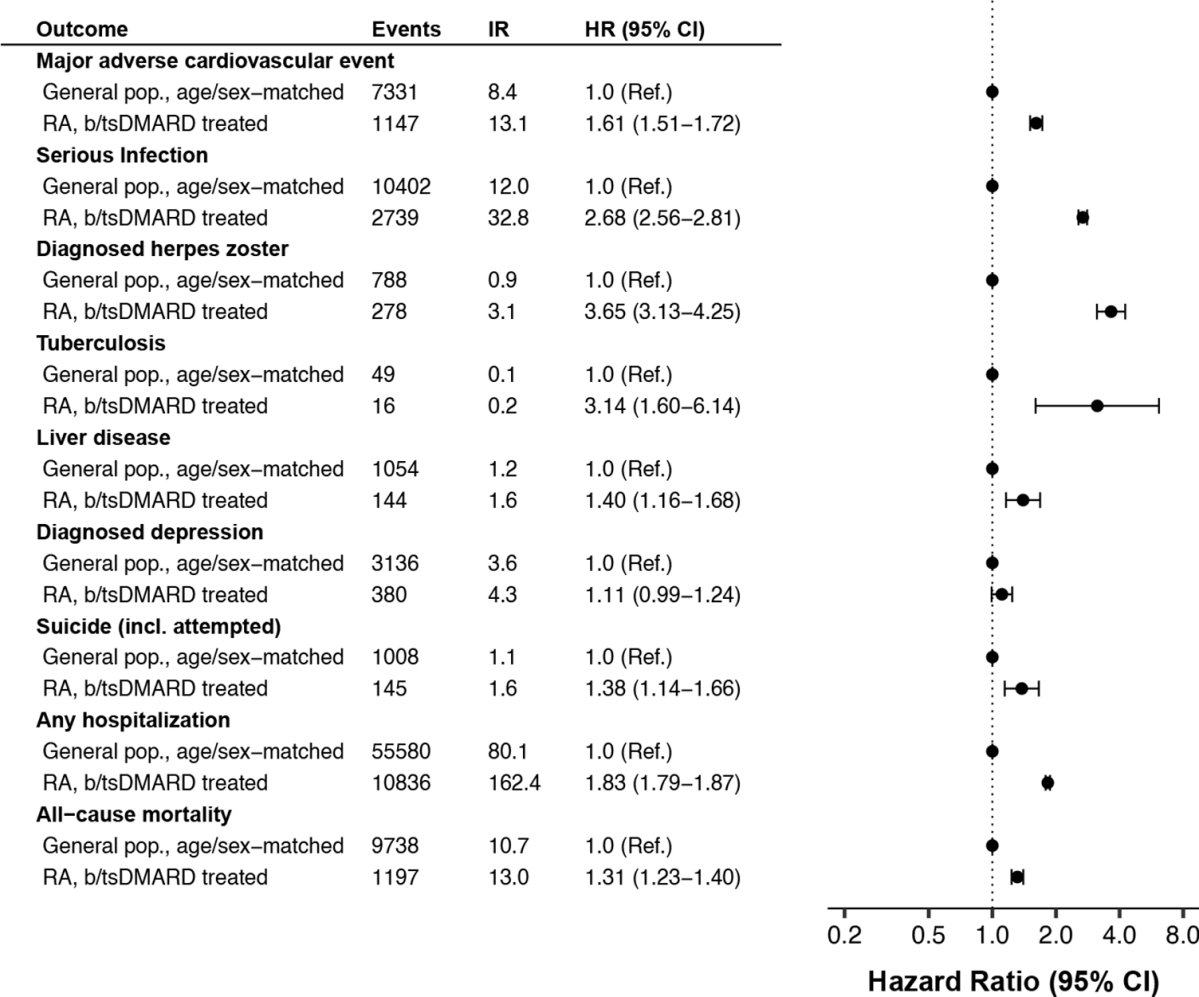
Incidence rate per 1000 person-years of selected safety outcomes, among all Swedish patients with RA who started b/tsDMARD 2010–2020, and a 5:1 age-sex matched general population sample, followed until 30 June 2021. HR, hazard ratio from Cox regression; RA, rheumatoid arthritis.

By contrast, the weighted rates of MACE was more similar across treatments, although a borderline significantly higher rate was seen on certolizumab pegol and rituximab versus etanercept. The pattern was similar for ACS and stroke (online supplemental figure S1). A lower rate of ACS was seen with baricitinib (vs etanercept), associated with broad confidence intervals (HR=0.42 (0.21–0.83)). Compared with the general population, patients with RA on b/tsDMARDs had a significant 60% higher rate of MACE (figure 3).

The difference to the general population rate was also pronounced for infections, with more than doubled rate for serious infections and more than tripled for herpes zoster among patients with RA on b/tsDMARDs. Infliximab and rituximab had about 30% higher rate of overall serious infections than etanercept, while the others had similar and non-significantly different rates. This differed from the pattern seen for herpes zoster as registered in specialty care, where the rate was almost four times higher on baricitinib and tofacitinib versus etanercept, but no other significant interdrug difference (vs etanercept) was observed. Though tuberculosis was rare, with only 16 events recorded across all treatment groups, this translated to a tripled rate compared with the general population (figure 3 and online supplemental table 5).

The weighted rate of non-steatosis liver disease was low (1–3 per 1000 PYs) across all b/tsDMARDs, about 40% higher than the rate seen in the general population (cf. figures 2 and 3). Similar patterns were seen for diagnosed depression (2–5 per 1000 PYs) and for (attempted) suicide (1–2 per 1000 PYs), about 10% and 40% higher than general population, respectively.

The weighted incidence rate of hospitalisation (for any cause) was 162 per 1000 PYs on b/tsDMARD, which was about 80% higher than the rate in the general population, with b/tsDMARDs. Significantly higher rates (vs etanercept) were observed on infliximab (18%), certolizumab pegol (15%) and rituximab (28%). The lowest rate was observed on tofacitinib (weighted HR=0.69 (0.49–0.96)).

All-cause mortality among patients with RA starting b/tsDMARD was about 30% higher than in the general population. Mortality rate was similar across TNFi, but about 30%–40% higher on the non-TNFi bDMARDs, with the numerically highest HR seen for baricitinib.

In age-sex standardised comparison to b/tsDMARD-naïve patients with RA (online supplemental figure S2), the b/tsDMARD-treated patients had significantly higher rates of serious infections, herpes zoster and tuberculosis, but slightly lower rate of MACE and diagnosed depression. Possibly indicative of substantial confounding, the b/tsDMARD-treated also had significantly lower rate of all-cause mortality (HR=0.65 (0.61–0.69)), cautioning us from drawing firm conclusions from the other rates.

### Analyses by adjustment method

While the weighting successfully balanced mean patient characteristics across the major treatment groups (online supplemental tables 3 and 4), it was not possible to reach acceptable balance for all variables in the two smallest groups (sarilumab, tofacitinib). Adjustment directly in multivariable Cox regressions gave very similar estimates throughout (online supplemental table 5). Due to differences in drug market entry, it was not possible to include an adjustment for year of treatment start in the weights. Similarly, availability of smoking data increased dramatically over time, which made it impossible to include in weight-models without losing balance in other covariates. Adjusting for year of treatment start and smoking in Cox models, however, left associations virtually unchanged (online supplemental table 5).

### Sensitivity analyses

Restricting the study period to the time after JAKi market entry reduced sample sizes drastically, and several differences between non-TNFi bDMARDs and etanercept were no longer significant, but it did not materially alter the comparison between the JAKi and etanercept (online supplemental figure S3). Ending follow-up at the onset of the COVID-19 pandemic had little impact on incidence rates or contrasts between bDMARDs, but the reduced sample size for sarilumab and JAKi excluded them from most comparative analyses for these groups (online supplemental figure S4).

## Discussion

This study followed close to 35 000 treatment initiations of b/tsDMARD among patients with RA in Sweden between 2010 and 2021 and estimated and compared the incidence of selected key safety outcomes between individual treatments while adjusting for a range of potential confounders. We found large differences in the rate of discontinuation of adverse events, several differences in rates of herpes zoster or overall serious infections and no clear difference in rate of MACE. Incident liver disease diagnoses and clinical depression were very rare in this cohort, independently of which b/tsDMARD was used.

These findings largely support previous reports and the currently established view of the relative safety of b/tsDMARDs. Largest differences were seen for the rate of discontinuation citing adverse events, where the ranked (highest to lowest) order we observed was similar to that by overall drug survival in a contemporary cohort of Danish patients with RA.^13^ It may in reality be difficult to assign a single cause for the choice to discontinue therapy (missing data can be substantial^14^) and a lower rate of discontinuation citing safety should not be directly interpreted as a better drug safety profile. We saw the lowest rate on rituximab, consistent with previous reports that this drug has longer overall drug survival than other b/tsDMARDs,^14 15^ despite a relatively higher rate of hospitalisation and serious infections. Also consistent with our data, previous reports found infliximab to have poorer drug survival and more discontinuation for adverse events compared with etanercept and adalimumab.^16 17^

With regard to cardiovascular risks, patients with RA starting etanercept b/tsDMARD were at increased risk of MACE compared with the matched general population comparators, a finding in line with previous studies.^18^ We also replicate previous findings of similar rates of ACS on different bDMARDs.^18 19^ In the ORAL surveillance trial of patients with RA with pre-existing cardiovascular risk factors, tofacitinib was associated with increased risk (vs TNFi) of MACE, at least beyond 18 months of follow-up.^20^ The low number of events on tofacitinib in the present material makes the result inconclusive but note that we did not observe any increased rate of MACE on baricitinib, where in fact the rate of ACS was significantly lower than on etanercept (possibly due to residual confounding). Venous thrombotic events were not included in this study, but the previously reported risk signal for JAKi was recently replicated in ARTIS, reporting age-standardised and sex-standardised IR of 5.2 per 1000 PYs on TNFi and 11.3 on JAKi (adjusted HR was 1.73 (1.24 to 2.42)).^10^ Another recent study from ARTIS compared overall cancer risks for patients with RA treat with b/tsDMARDs to the general population and found no increased risks for TNFi, rituximab or tocilizumab, a possible risk increase for abatacept (HR=1.3 (1.1–1.6)), and too limited follow-up for a comparison to the JAKi.^11^

It is well established that patients treated with b/tsDMARDs have an increased rate of serious infections and reactivation of latent varicella-zoster compared with the general population.^21–23^ A particularly increased rate of herpes infections and herpes zoster has been reported in RCTs of JAKi in RA.^24^ Compared with etanercept, we observed a more than tripled rate of herpes zoster on JAKi, which is similar to estimates from the German RABBIT register,^25^ and in line with a meta-analysis of randomised trials.^24^ Also consistent with these studies, the rate of other serious infections was not higher on JAKi versus etanercept. A higher rate of serious infections on rituximab versus other bDMARDs has been suggested by some previous studies^21^ and has been reported compared with other disease modifying drugs in multiple sclerosis.^26^ In accordance with some previous reports,^27 28^ we also observed a significantly increased rate of serious infections on infliximab versus etanercept.

Increased rates of hepatobiliary adverse events have been noted in some b/tsDMARD RCTs in RA, for example, of the etanercept biosimilar SB4.^29^ Similar to previous studies,^30^ the rate of liver disease was very low in our cohort, regardless of b/tsDMARD used, and we noticed no safety signal for this outcome. Similarly, there has been interest, including from regulatory agencies, in a possible effect of infliximab on the risk of depression and attempted suicide.^31 32^ We did not observe any significant differences in the rate of diagnosed depression or attempted suicide by b/tsDMARD.

Finally, we observed several significant differences in the rate of all-cause hospitalisation and mortality. First, we found an overall modestly increased mortality in b/tsDMARD-treated RA compared with the general population (31% increase). Some evidence suggests an improving mortality in RA over recent decades,^33 34^ and to ensure maximal relevance for contemporary clinical practice, we restricted our study to patients initiating b/tsDMARD therapies during the most recent ten (instead of maximally 24) years. We also observed several significant differences between b/tsDMARDs. These differences are difficult to interpret, reflecting a combination of possibly true and drug-related risk differences for a range of adverse events and the residual confounding by factors not adequately controlled for. It is possible that surveillance linked to the mode of administration also influence the likelihood to be hospitalised; drugs given through infusion had highest rates of hospitalisations (rituximab and infliximab), while lowest were seen for the oral JAKi. We note that rituximab was significantly associated with higher rates of both hospitalisation and mortality, in fact, associated with higher rates of all outcomes defined by hospitalisation. But it should also be noted that the confounder-adjustment markedly reduced these associations; the non-specificity of this increased risk may itself be a signal of residual confounding. This highlights the degree of channelling bias between these groups in the real-world clinical setting, where, as the crude incidence rates show, it would indeed be correct to say patients initiating some drugs are at a higher rate of serious adverse events without implying a risk increase conferred by the drug itself.^35^

Strengths of this study include the national coverage, giving a large cohort and avoiding selection bias and the collection of safety outcomes independently from drug assignment through national registers with an established high validity. We were able to simultaneously include all b/tsDMARDs used in clinical practice and could accommodate a broad range of possible confounding factors. We could further demonstrate that the method of adjustment (propensity weighting vs multivariable Cox regression) did not influence the findings of the study. For JAKi’s in particular, our results add to a relatively limited evidence-base.

Our study also has several limitations. We used a common study design, including a shared model to adjust for confounding, across all outcomes. This allowed a streamlined analysis and simplifies the comparison across outcomes, but is more susceptible to residual confounding and other biases which a bespoke study design for each outcome might have avoided. That said, our set of covariates included a wealth of potential risk factors that are shared across outcomes and also markers of general frailty. Due to the multitude of statistical tests, several false positive findings may be expected. Despite the large, national cohort, the scarcity of TB, liver disease and suicide made these results inconclusive, with broad confidence intervals.

This study was only possible thanks to the well-established Swedish clinical register SRQ, and the ARTIS safety monitoring programme. The first decade of bDMARD therapy in Sweden was summarised by Simard *et al* in 2011,^36^ emphasising the importance of patient characteristics when evaluating clinical outcomes. Together, studies from ARTIS have now provided relevant clinical data covering an observation period of over twenty years. This demonstrates that a register-initiative initiated and maintained by the clinical profession can persist over time and continue to provide important drug safety data, meeting the needs of healthcare and the pharmaceutical industry and regulatory agencies.^37^ The experience from PASS studies of bDMARDs has been important for the development of efficient use of risk management plans in the European regulatory system.^38 39^ Data from ARTIS and other similar registries, like the Danish DANBIO,^40^ RABBIT in Germany^41^ and BSRBR in the UK,^42^ have been instrumental in the required postmarketing characterisation of long-term safety profiles for b/tsDMARDs.

In conclusion, this study provides a comprehensive assessment of safety outcomes of particular interest, for most b/tsDMARDs available for the treatment of RA. Our results corroborate and extend previous evidence that the currently available b/tsDMARDs have acceptable and on the whole similar safety profiles, but that differences exist in particular concerning tolerability, specific infection risks and for specific serious but rare outcomes. To inform risk-benefit trade-offs, these data on safety outcomes should be combined with corresponding data on effectiveness. We believe that studies which, like the current one, can include data on all used treatment options in one cohort are particularly valuable to avoid problems with generalisability across studies. While such effectiveness studies do exist,^10 15 18 25^ both overall and in defined subsets of patients, they (similar to the situation for safety data) represent a minority of all publications on this topic. Finally, although data on safety and effectiveness of this kind are a necessary foundation for clinical decision-making, the value-based decision on what ratio of safety concerns and treatment benefits defines the ‘best’ treatment choice should reside with the individual patient and treating rheumatologist.

## Data Availability

Data may be obtained from a third party and are not publicly available. The data was retrieved from national Swedish registers, which can only be accessed with specific permissions from the Swedish register-holding authorities.
