# Supplementary material for: Safety of biological and targeted synthetic disease-modifying antirheumatic drugs for rheumatoid arthritis as used in clinical practice: results from the ARTIS programme
Source: Ann Rheum Dis. 2023 Feb 14;82(5):601–10. doi: 10.1136/ard-2022-223762 (PMC10176333; doi:10.1136/ard-2022-223762)

Online Supplement for

Safety of b/tsDMARDs for RA as used in clinical practice - results from the ARTIS program

Thomas Frisell,[1] PhD, Hannah Bower[1], PhD, Matilda Morin[1], MSc, Eva Baecklund,[2] MD/PhD, Daniela Di Giuseppe, [1] PhD, Bénédicte Delcoigne,[1] PhD, Nils Feltelius,[3] MD/PhD, Helena Forsblad-d'Elia,[4] MD/PhD, Elisabet Lindqvist,[5,6] MD/PhD, Ulf Lindström,[4] MD/PhD, Johan Askling,[1,7] MD/PhD on behalf of the ARTIS Study group

[1]Clinical Epidemiology Division, Department of Medicine, Solna, Karolinska Institutet, Stockholm, Sweden [2] Department of Medical Sciences, Rheumatology, Uppsala University, Sweden. [3] Department of Public Health and Caring Sciences, Uppsala University, Uppsala, Sweden [4] Department of Rheumatology and Inflammation Research, Institute of Medicine, Sahlgrenska Academy at University of Gothenburg, Gothenburg, Sweden. [5]Section of Rheumatology, Department of Clinical Sciences Lund, Lund University, Lund, Sweden. [6]Department of Rheumatology, Skåne University Hospital, Lund, Sweden[7]Rheumatology, Theme Inflammation and Ageing, Karolinska University Hospital, Stockholm, Sweden

Contents

Extended methods supplement 2
The ARTIS surveillance program for immunomodulatory drugs against chronic inflammatory arthritis 2
Data sources 2
Setting 2
The Swedish Rheumatology Quality Register (SRQ) and ARTIS 3
The National Patient Register 3
The Prescribed Drug Register 4
The National Cancer Register. 4
The Cause of Death Register 4
The Tuberculosis Register 4
The Total Population Register 5
Supplemental references 5
Supplemental table 1: Outcome definitions 7
Supplemental table 2: Covariate definitions 9
Supplemental Results 10
Supplemental table 3. Post-weighting patient characteristics at start of b/tsDMARD therapy, among all Swedish RA patients, 2010-2020 10
Supplemental table 4. Post-weighting population standardized bias 11
Supplemental table 5. Comparison of hazard ratios from crude, multivariable, and weighted Cox regression 12
Figure S1. MACE component outcomes among all Swedish RA patients who started b/tsDMARD 2010-2020. 15
Figure S2. Incidence rate of safety outcomes comparing Swedish RA patients who started b/tsDMARD 2010-2020 to b/tsDMARD-naive patients with RA. 16
Figure S3. Crude and weighted incidence rate per 1000 person-years of selected safety outcomes by b/tsDMARD, and adjusted hazard ratios versus etanercept, among all Swedish RA patients who started treatment *2017-2020*, from JAKi market entry, followed until 30 June 2021 17
Figure S4. Crude and weighted incidence rate per 1000 person-years of selected safety outcomes by b/tsDMARD, and adjusted hazard ratios versus etanercept, among all Swedish RA patients who started treatment 2010-2020, followed *until the COVID-19 pandemic, 28 Feb 2020*. 18

Extended methods supplement

The ARTIS surveillance program for immunomodulatory drugs against chronic inflammatory arthritis

In Sweden, the first biologic disease modifying anti-rheumatic drugs (bDMARDs) were made available for the treatment of RA in 1999. At that time, the ARTIS (Anti-Rheumatic Treatment in Sweden) monitoring system was started in collaboration with the Swedish Medical Products Agency, and serves as a national surveillance program for investigation of safety and effectiveness of new anti-rheumatic drugs. The ARTIS database is constructed by regular (often annual) extractions of data from the Swedish Rheumatology Quality Register (SRQ), linked to data from other national Swedish registers, as depicted in the figure below and further described under Data sources.

Figure. Schematic illustration of the ARTIS study database

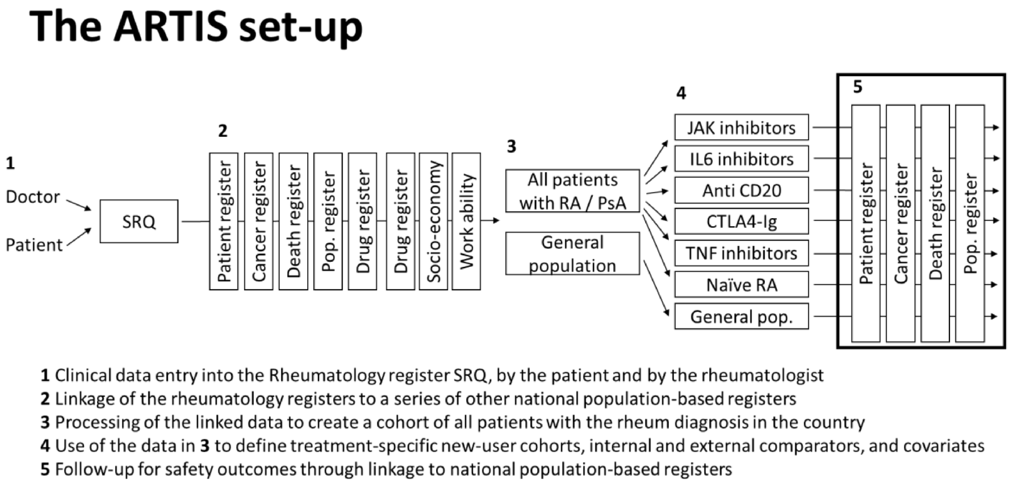

The ARTIS study database is built by identifying an index population of patients with chronic inflammatory joint diseases in Sweden and a matched general population comparator cohort, with information on patient characteristics, safety and treatment effectiveness outcomes retrieved through national registers.

Data sources

Setting

Swedish health-care is tax-funded and offers universal access. Hospital referral is based on geography rather than insurance-status. Patients with inflammatory arthritides are typically treated by rheumatologists, the vast majority of whom work in public and hospital-based clinics. Health and demographic information is recorded in a series of registers with a very high degree of completeness resulting from the mandatory and semi-automated registration. Based on each Swedish resident's unique personal identification number, issued to all Swedish residents alive in 1947 or born thereafter, linkage of data from different registers is possible.[1] The registers are maintained by governmental bodies (the main registers used in this project are

held by the National Board of Health and Welfare (Socialstyrelsen) and Statistics Sweden), who may perform data linkages and provide de-identified data for research purposes.

The Swedish Rheumatology Quality Register (SRQ) and ARTIS

The Swedish Rheumatology Quality Register (SRQ) was started in 1995 by the Swedish Rheumatology Society to improve the healthcare and treatment for patients with RA. SRQ followed on regional register initiatives, to enable a national real-world documentation of many different aspects of RA and developed over time into a harmonized national register. SRQ was started mainly for patients with RA, but over time it has been expanded to cover several other rheumatic diseases including ankylosing spondylitis and psoriatic arthritis, Currently, some 29,000 patients with RA initiating over 70,000 biological treatments have been registered in SRQ. The register also contains early RA patients, and increasingly, RA patients can be followed from their first RA diagnosis and onwards. In conjunction with each patient visit, the treating rheumatologist enters data on disease activity and anti-rheumatic treatment, and the patient enters data on symptoms and health-status. The coverage of the SRQ is high, and was in 2015 estimated to be about 90% for new initiations of bDMARDs among patient with RA in Sweden.[2]

The National Patient Register

The Swedish National Patient Register collects information on all hospitalized (inpatient treated) patients, and all visits to physicians in non-primary outpatient care (such as a visit to a rheumatologist).[3] Diagnoses are assigned by the discharging physician, and coded according to the ICD, with version 8 used until 1986, version 9 from 1987 to 1996, and version 10 since 1997. The register also collects information of the treating hospital, medical specialty, medical procedures or interventions, and dates of hospitalization and discharge. The inpatient component was originally initiated by several counties in 1964, had 85% country-wide coverage in 1983, and is considered complete since 1987. The outpatient component of the Patient register was initiated with nationwide coverage in 2001, but the coverage was poor for several medical areas in the first years, with substantial missingness in e.g. diagnosis. This improved quickly in the first five years of the outpatient component, and since 2010, the missingness in diagnosis is stable about 1% for inpatient care and 3% for outpatient care.

Reporting data to the register is mandatory for all health care providers in Sweden, and was done on an annual basis until 2015, when the frequency increased to monthly. Data is mostly reported by Sweden’s 21 Regions (the intermediate governmental level, responsible for providing public health care), who have established different local processes for extracting the mandatory information from their regional electronic medical records system, and uploading it through the National Board of Health and Welfare’s secure file transfer protocol. About 1% of inpatient healthcare visits and 5% of outpatient healthcare visits are reported directly from private healthcare providers (this can be privately funded healthcare, or because the private provider for some other reason does not share electronic medical records with the healthcare Region). The local files are combined and subjected to automated and semi-automated checks of logical consistency and content deviations from e.g. expected frequency of healthcare visits.

Due to the automated processes for extracting and reporting data, the register is today expected to correspond well to the information recorded in the local medical records. Historically, validation against medical files have found an overall error rate in the main diagnoses of 4% at the ICD chapter level, and 12% at the three digit level.[4] Diagnoses recorded in the medical record can of course also be incorrect, and chart reviews and validation of the RA diagnosis based on different algorithms applied to the register data indicate a positive predictive value for a register-based diagnosis of RA around 90%.[5-7]

The Prescribed Drug Register

Started in July 2005, the National Prescribed Drug Register contains data on all dispensations of prescription medication at Swedish pharmacies. The data reporting is mandatory, and linked to the softwares used in all pharmacies that dispenses regulated (i.e., prescription) medication. Data is uploaded daily to the Swedish eHealth Agency, who sends monthly summaries to the National Board of Health and Welfare where the register is finalized. The register contains information on the prescription (who wrote it, when, who is filling it, and was there a note on dosage made) and on the dispensed item (the package ID, the number of packages). The package ID is according to a national list and can be translated to the brand name, ATC code, mode of delivery, amount of drug, and cost of drug. Due to the automated set-up, the register is considered completely accurate. The register does not capture the indication for treatment, and will not cover medications used in a hospital setting (as they are not dispensed from pharmacies) or which are bought at pharmacies without prescriptions (i.e., over the counter drugs).

The National Cancer Register.

The Swedish National Cancer Register was established in 1958 and contains information on date of cancer (and some selected pre-cancers) onset, and type of cancer according to the ICD classification and morphology/histology. About 99% of cancers have been morphologically verified. Reporting of incident cancers (including invasive malignancies as well as selected types of cancer in situ) is mandatory and semi-automated, resulting in an estimated coverage greater than 95%.[8,9] In practice, reporting to the Cancer register often required two complementary reports: a pathologists report of morphological characteristics and a clinican’s report of clinical stage and ICDO-coded diagnosis. As soon as one of these reports is sent to the Cancer register’s regional center, it will start a process of investigation with reminders sent out until the report can be completed, and only then is it formally recorded in the register. The pathology reports are sent directly from the medical records system used at clinical laboratories, and have little lag time and high degree of completeness, while the clinical report can have substantial lag. For cancers which are never biopsied, this means that the coverage is much less complete. This is known to lead to underestimates of internal cancers with poor prognosis, in particular in older patients, and as much as 30% of pancreatic cancer has been estimated to be missing from the Cancer register.[10,11] For such types of cancer, it is recommended to also use data from the Cause of Death register.

The Cause of Death Register

The National Cause of Death Register contains information on date and cause of death (underlying and contributory) for all deceased residents, including deaths among Swedish residents who died abroad. The register was started in 1952, and the data is considered complete since 1961.[12] From that year and onward, cause of death is missing for less than 0.5% of deceased individuals, and in 2002, a validation study estimated that only 3.3% had any errors at the three-digit level of the ICD-coded underlying cause of death. The register depends on a semi-manual process of data collection, where the medical doctor who signs the death certificate is responsible for uploading the causal chain leading to the death directly to the National Board of Health and Welfare, where coding consistency is checked and follow-up questions can be sent out to confirm or complete records. The high completeness of the register is possible since the Board have access to the quickly updated census information, and send out reminders and follow-up questions for each recorded death lacking a valid cause of death certificate.

The Tuberculosis Register

The Public Health Agency maintains a register of certain communicable diseases, including a sub register on tuberculosis. This *Tuberculosis (TB) Register* started in 1969 on a national level and contains data on active TB diagnoses. Reporting incident TB cases to this register is done in parallel by both the microbiological laboratories and clinicians when a patient is culture-positive. In addition, any individual

clinically diagnosed with TB is reported to the TB register's web-based reporting system by the clinician. Completeness and quality of the data retrieved is monitored weekly, resulting in 100% coverage of all cases verified by culture.[13,14]

The Total Population Register

The Total Population Register is maintained by Statistics Sweden as the backbone of the Swedish system of national register.[15] Derived from the continuously updated census data at the Swedish Taxation Office, the register lists data on residency at a given point in time since it was founded in 1961, and dates of emigration/immigration for all subjects ever resident in Sweden since 1961. This register is used to identify the general population comparison cohorts, and to censor subjects who die or emigrate during follow-up.

Supplemental references

1. Ludvigsson JF, Otterblad-Olausson P, Pettersson BU, Ekbom A. The Swedish personal identity number: possibilities and pitfalls in healthcare and medical research. *European journal of epidemiology.* 2009;24(11):659-667.
2. Wadstrom H, Eriksson J, Neovius M, Askling J, On Behalf Of The Artis Study G. How good is the coverage and how accurate are exposure data in the Swedish Biologics Register (ARTIS)? *Scandinavian journal of rheumatology.* 2015;44(1):22-28.
3. Socialstyrelsen. Det statistiska registrets framställning och kvalitet - Patientregistret. 2022; https://www.socialstyrelsen.se/globalassets/sharepoint-dokument/artikelkatalog/statistik/2022-2-7767.pdf.
4. Socialstyrelsen. Inpatient Diseases in Sweden 1987-2008 [in Swedish]. 2008.
5. Eriksson JK, Neovius M, Ernestam S, Lindblad S, Simard JF, Askling J. Incidence of rheumatoid arthritis in Sweden: a nationwide population-based assessment of incidence, its determinants, and treatment penetration. *Arthritis Care Res (Hoboken).* 2013;65(6):870-878.
6. Knight A, Sandin S, Askling J. Risks and relative risks of Wegener's granulomatosis among close relatives of patients with the disease. *Arthritis and rheumatism.* 2008;58(1):302-307.
7. Waldenlind K, Eriksson JK, Grewin B, Askling J. Validation of the rheumatoid arthritis diagnosis in the Swedish National Patient Register: a cohort study from Stockholm County. *BMC musculoskeletal disorders.* 2014;15:432.
8. Barlow L, Westergren K, Holmberg L, Talbäck M. The completeness of the Swedish Cancer Register: a sample survey for year 1998. *Acta oncologica (Stockholm, Sweden).* 2009;48(1):27-33.
9. Mattsson B, Wallgren A. Completeness of the Swedish Cancer Register. Non-notified cancer cases recorded on death certificates in 1978. *Acta radiologica Oncology.* 1984;23(5):305-313.
10. Lambe M, Wigertz A, Sandin F, et al. Estimates of lung and pancreatic cancer survival in Sweden with and without inclusion of death certificate initiated (DCI) cases. *Acta oncologica (Stockholm, Sweden).* 2020;59(11):1322-1328.
11. Socialstyrelsen. Bortfall i cancerregistret vid jämförelse med dödsorsaksintyg. 2021; https://www.socialstyrelsen.se/globalassets/sharepoint-dokument/artikelkatalog/statistik/2021-9-7530.pdf.
12. Socialstyrelsen. Det statistiska registrets framställning och kvalitet - Dödsorsaksregistret. 2022; https://www.socialstyrelsen.se/globalassets/sharepoint-dokument/artikelkatalog/statistik/2022-5-7909.pdf.
13. Inghammar M, Ekbom A, Engström G, et al. COPD and the risk of tuberculosis--a population-based cohort study. *PLoS One.* 2010;5(4):e10138.
14. Arkema EV, Simard JF. Cohort profile: systemic lupus erythematosus in Sweden: the Swedish Lupus Linkage (SLINK) cohort. *BMJ open.* 2015;5(8):e008259.

15. Ludvigsson JF, Almqvist C, Bonamy AK, et al. Registers of the Swedish total population and their use in medical research. *European journal of epidemiology.* 2016;31(2):125-136.

Supplemental table 1: Outcome definitions

Outcome	Data source	Codes
**Treatment stop due to adverse event**	SRQ	The recorded reason for discontinuation. Discontinuation listing adverse event or safety as reason. All other reasons, or missing, were considered censoring events.
**Acute coronary syndrome**	Patient register: in- and outpatient, main or secondary diagnosis<br><br>Causes of death register: main or contributing cause of death	I20.0, I21, I22
**Stroke**	Patient register: in- and outpatient, main or secondary diagnosis<br><br>Causes of death register, main or contributing cause of death	I60-I64
**Serious infection**	Patient register: Main diagnosis in inpatient care	A00 - B99<br>D733<br>E060 E321<br>G00 G01 G02 G042 G050 G051 G052 G06 G07<br>H000 H010 H030 H031 H061 H10 H130 H131 H160 H162 H190 H191 H192 H220 H30 H440 H600 H601 H603 H620 H621 H622 H623 H66 H670 H671 H68 H70 H730 H750 H940<br>I301 I330 I39 I400 I410 I411 I412 I430 I520 I521 I681 I980 I981<br>J00 J01 J02 J03 J04 J05 J06 J09 J10 J11 J12 J13 J14 J15 J16 J170 J171 J172 J173 J178 J18 J20 J21 J22 J32 J340 J36 J37 J383F J383R J390 J391 J440 J85 J86<br>K044 K045 K046 K047 K050 K052 K102 K113 K122 K140 K230 K35 K570 K572 K574 K578 K61 K630 K650 K67<br>L00 L01 L02 L03 L04 L05 L08 L303<br>M00 M01 M03 M462 M463 M465 M490 M491 M492 M493 M600 M630 M631 M632 M650 M651 M710 M711 M726 M86 M900 M901 M902<br>N080 N088 N10 N11 N136 N151 N159 N160 N290 N291 N300 N308 N33 N340 N341 N342 N390 N412 N431 N450 N481 N482 N49 N51 N61 N700 N710 N72 N730 N733 N74 N751 N760 N762 N770 N771<br>O070 O075 O080 O23 O353 O411 O753 O85 O86 O91 O98
**Tuberculosis**	The Public Health Agency's register of communicable diseases.	N/A

	Mandatory registration of laboratory confirmed cases.	
**Herpes Zoster**	Patient register: in- and outpatient, main or secondary diagnosis	B01, B02
**Diagnosed depression**	Patient register: in- and outpatient, main or secondary diagnosis	F32-F33
**Suicide (including attempted suicide)**	Patient register: External cause codes in inpatient care<br><br>Causes of death register, main or contributing cause of death	X60-X84, Y10-Y34
**Non-steatosis diseases of liver**	Patient register: in- and outpatient, main or secondary diagnosis<br><br>Causes of death register, main or contributing cause of death	K70-K77, except K76.0
**Any hospitalization**	Patient register	Any overnight hospital stay
**All-cause mortality**	Register of the total population.	Any listed date of death

Supplemental table 2: Covariate definitions

Demographics			
**Variable**	**Data source**	**Type/Parameterization**	**Defined at time**
**Age**	From national ID	Second degree polynomial	Treatment start
**Sex**	From national ID	Binary: Male/female	Last recorded
**Country of birth**	Register of total population	Binary: Swedish/non-Swedish	Time fixed
**Education level**	LISA	Categorical: <9 yrs, 9-12 yrs, 12+ yrs	Year before treatment start
**Smoking**	SRQ (patient-reported)	Binary: Ever (current or former) vs Never	Closest to treatment start, up to two years after
Clinical characteristics of RA			
**Variable**	**Data source**	**Type/Parameterization**	**Defined at time**
**Seropositive RA**	SRQ	Binary: by diagnosis in SRQ, if RF+ or ACPA+ then Yes, else No	Treatment start
**RA duration**	SRQ	Second degree polynomial	Treatment start
**Number of previous b/tsDMARDs**	SRQ	Categorical: 0, 1-2, 3+	Counting all before treatment start
**DAS28CRP**	SRQ	Second degree polynomial	Treatment start
**HAQ**	SRQ	Second degree polynomial	Treatment start
**Co-medication with methotrexate**	SRQ	Binary: yes vs no	Treatment start
**Co-medication with other csDMARD**	SRQ	Binary: yes vs no	Treatment start
**Co-medication with corticosteroids**	SRQ	Binary: yes vs no	Treatment start
**Days hospitalized**	The Patient register	Categorical: 0, 1-9, 10+	5 yrs before treatment start
Definitions of baseline diseases considered as potential confounders (all binary: yes vs no)			
**Disease**	**Data source**	**ICD10 or NOMESCO**	**Look-back time**
**Malignancy**	The Cancer register	All except benign tumors	10 yrs
**Infection**	As the outcome	As the outcome	5 yr
**Herpes zoster**	As the outcome	As the outcome	5 yr
**Knee or hip prosthesis**	Procedure codes from the Patient register	NGB, NFB	10 yrs
**Chronic pulmonary disease**	The Patient Register	J41-J44	5 yrs
**Diabetes**	The Patient Register	E10-E14, O24	5 yrs
**CVD**	ACS or stroke, per outcome definitions	ACS or stroke, per outcome definitions	5 yrs
**Depression**	As the outcome	As the outcome	5 yrs

Supplemental Results

Supplemental table 3. Post-weighting patient characteristics at start of b/tsDMARD therapy, among all Swedish RA patients, 2010-2020

	ETA	ADA	INF	CTZ	GOL	ABA	RTX	TCZ	SAR	BAR	TOF
**Demographics**											
Age, mean	58	58	57	58	58	58	58	58	60	59	57
Female	77%	78%	76%	78%	78%	77%	78%	78%	79%	79%	74%
Highest education, 9y or less	11%	11%	11%	11%	11%	11%	11%	11%	11%	12%	9%
Highest education, 10y to 12y	58%	59%	59%	59%	58%	58%	58%	59%	58%	56%	57%
Highest education, >12y	31%	31%	30%	31%	31%	31%	30%	30%	32%	32%	34%
Swedish-born	85%	86%	85%	85%	85%	85%	85%	86%	88%	84%	85%
**RA clinical characteristics**											
Rheumatoid factor positive	75%	75%	73%	75%	74%	75%	75%	75%	75%	74%	69%
Disease duration, yrs, mean	12.9	12.7	12.3	12.7	12.8	12.5	13.2	12.3	12.7	13.4	11.8
DAS28	4.4	4.4	4.3	4.4	4.4	4.4	4.3	4.4	4.4	4.3	4.3
HAQ	1.1	1.1	1.1	1.1	1.1	1.1	1.1	1.1	1.2	1.1	1.1
Conc. MTX	56%	56%	57%	56%	56%	56%	55%	55%	51%	53%	49%
Conc. non-MTX csDMARD	14%	15%	16%	15%	15%	15%	15%	13%	10%	13%	12%
Conc. oral steroids	46%	46%	46%	46%	47%	45%	48%	44%	46%	43%	42%
Prior b/tsDMARDs											
0	44%	44%	44%	44%	43%	43%	40%	42%	28%	39%	34%
1-2	39%	39%	41%	39%	38%	40%	41%	40%	49%	43%	46%
3+	18%	18%	15%	18%	19%	18%	20%	18%	22%	19%	21%
**Medical history***											
Malignancy	5%	5%	5%	5%	5%	5%	5%	5%	3%	5%	5%
Serious infection	12%	12%	12%	12%	12%	12%	13%	11%	16%	12%	11%
Serious herpes zoster	1%	1%	1%	1%	1%	1%	1%	1%	0%	1%	1%
Joint surgery	11%	10%	9%	11%	11%	11%	11%	10%	11%	11%	10%
COPD	4%	3%	3%	4%	4%	4%	4%	4%	4%	3%	4%
Diabetes mellitus	8%	8%	7%	8%	8%	8%	8%	7%	8%	8%	8%
ACS	2%	2%	2%	2%	2%	2%	2%	3%	2%	2%	2%
Stroke	1%	1%	1%	1%	1%	1%	1%	1%	0%	1%	0%
Days hospitalized											
0	68%	70%	68%	69%	69%	68%	68%	70%	71%	70%	74%
1-9	18%	18%	20%	18%	18%	19%	19%	17%	15%	17%	17%
10+	14%	13%	12%	13%	13%	13%	13%	13%	14%	13%	10%

Notes: *) Medical history in five years before treatment start, except Serious infection (one year before start) and malignancy or joint surgery (ten years before)

Supplemental table 4. Post-weighting population standardized bias

	ETA	ADA	INF	CTZ	GOL	ABA	RTX	TCZ	SAR	BAR	TOF
**Demographics**											
Age, mean	0.01	-0.02	-0.06	-0.01	0.00	0.00	0.03	0.00	0.12	0.07	-0.08
Female	0.00	0.01	-0.04	0.00	0.00	-0.01	0.01	0.02	0.03	0.04	-0.09
Highest education, 9y or less	0.00	-0.01	-0.01	-0.01	0.02	0.00	0.02	0.00	-0.01	0.02	-0.07
Highest education, 10y to 12y	0.00	0.01	0.02	0.00	0.00	0.00	0.00	0.01	-0.01	-0.04	-0.03
Highest education, >12y	0.00	0.00	-0.01	0.00	-0.01	0.00	-0.01	-0.01	0.02	0.03	0.07
Swedish-born	0.00	0.02	-0.01	0.01	-0.02	0.00	-0.02	0.02	0.09	-0.05	-0.01
**RA clinical characteristics**											
Rheumatoid factor positive	0.01	0.01	-0.05	0.01	0.00	0.00	0.01	0.00	0.01	-0.02	-0.13
Disease duration, yrs, mean	0.01	0.00	-0.04	-0.01	0.00	-0.03	0.04	-0.04	0.00	0.06	-0.09
DAS28	0.00	0.00	-0.01	0.00	0.01	0.01	-0.01	0.02	0.05	-0.03	-0.08
HAQ	0.00	-0.01	-0.02	-0.01	0.01	0.00	0.02	0.03	0.07	0.02	-0.07
Conc. MTX	0.01	0.01	0.03	0.01	0.00	0.01	-0.01	-0.03	-0.10	-0.07	-0.14
Conc. non-MTX csDMARD	0.00	0.00	0.04	0.01	0.01	0.01	0.01	-0.04	-0.13	-0.04	-0.07
Conc. oral steroids	0.00	0.01	0.01	0.00	0.01	-0.02	0.05	-0.05	0.01	-0.06	-0.08
Prior b/tsDMARDs											
0	0.02	0.03	0.02	0.02	0.02	0.01	-0.06	-0.01	-0.29	-0.08	-0.18
1-2	-0.02	-0.02	0.03	-0.01	-0.04	0.00	0.02	0.00	0.20	0.06	0.13
3+	-0.01	-0.01	-0.07	-0.01	0.03	-0.01	0.04	0.01	0.11	0.02	0.07
**Medical history[a]**											
Malignancy	0.01	0.00	0.00	-0.01	-0.01	0.01	0.01	-0.02	-0.10	0.01	0.02
Serious infection	0.01	0.00	0.00	-0.01	-0.02	0.00	0.01	-0.02	0.12	0.00	-0.04
Serious herpes zoster	-0.01	0.01	0.01	0.00	0.00	-0.01	0.01	0.00	-0.05	-0.01	0.05
Joint surgery	0.01	-0.01	-0.04	0.00	0.02	0.01	0.01	0.00	0.01	0.02	-0.02
COPD	0.01	-0.02	-0.01	-0.01	0.00	0.02	0.00	0.00	0.04	-0.02	0.03
Diabetes mellitus	0.00	-0.01	-0.03	0.01	0.01	0.01	0.02	-0.02	0.01	0.00	0.00
ACS	0.01	-0.02	0.00	0.01	0.00	-0.01	-0.01	0.03	-0.02	0.01	-0.03
Stroke	0.01	-0.01	0.01	0.00	0.01	0.01	0.01	0.00	-0.08	-0.01	-0.08
Days hospitalized											
0	-0.02	0.02	-0.01	0.02	0.00	-0.01	-0.02	0.02	0.05	0.02	0.11
1-9	0.00	-0.01	0.04	0.00	-0.01	0.01	0.01	-0.03	-0.08	-0.02	-0.04
10+	0.02	-0.02	-0.04	-0.02	0.00	0.01	0.01	0.00	0.02	0.00	-0.10

Notes: a) Medical history in five years before treatment start, except serious infection (one year before start) and malignancy or joint surgery (ten years before). Standardized bias larger than 0.1 was considered indicative of poor balance, marked in red.

Supplemental table 5. Comparison of hazard ratios from crude, multivariable, and weighted Cox regression

Outcome	Events	IR/1000 PYR	Crude HR	Multivariable[a] Cox HR	Multivariable[a] + start year and smoking Cox HR	IPTW[a] Cox HR
**Stop due to Adverse event**						
**Etanercept**	446	18.0	1.0 (Ref.)			
**Adalimumab**	348	27.9	1.46 (1.27-1.68)	1.15 (1.00-1.32)	1.17 (1.02-1.35)	1.07 (0.92-1.25)
**Infliximab**	99	12.5	0.69 (0.55-0.85)	1.23 (0.99-1.54)	1.22 (0.98-1.53)	1.25 (0.99-1.58)
**Certolizumab pegol**	203	33.9	1.96 (1.66-2.32)	1.73 (1.46-2.04)	1.70 (1.44-2.00)	1.53 (1.28-1.82)
**Golimumab**	191	32.7	1.93 (1.63-2.29)	1.51 (1.28-1.79)	1.45 (1.22-1.71)	1.44 (1.19-1.73)
**Abatacept**	459	55.2	2.90 (2.56-3.29)	1.19 (1.04-1.36)	1.19 (1.04-1.36)	1.18 (1.03-1.36)
**Rituximab**	328	21.8	1.30 (1.14-1.50)	0.66 (0.58-0.77)	0.65 (0.56-0.75)	0.67 (0.57-0.78)
**Tocilizumab**	241	31.1	1.77 (1.52-2.07)	0.71 (0.61-0.83)	0.70 (0.60-0.82)	0.72 (0.60-0.85)
**Sarilumab**	30	94.7	3.45 (2.38-5.01)	1.18 (0.82-1.71)	1.32 (0.90-1.91)	1.25 (0.79-1.98)
**Baricitinib**	114	39.0	1.62 (1.32-1.98)	0.57 (0.46-0.70)	0.63 (0.50-0.78)	0.57 (0.45-0.73)
**Tofacitinib**	56	97.6	4.03 (3.06-5.31)	1.30 (0.98-1.72)	1.40 (1.05-1.86)	1.25 (0.85-1.84)
**Major adverse cardiovascular event**						
**Etanercept**	240	10.1	1.0 (Ref.)			
**Adalimumab**	145	12.2	1.22 (0.99-1.50)	1.24 (1.01-1.53)	1.25 (1.01-1.54)	1.12 (0.89-1.41)
**Infliximab**	86	11.3	1.13 (0.88-1.44)	1.04 (0.81-1.33)	1.05 (0.82-1.36)	1.00 (0.72-1.39)
**Certolizumab pegol**	76	13.2	1.31 (1.02-1.70)	1.42 (1.10-1.82)	1.41 (1.09-1.83)	1.31 (0.99-1.73)
**Golimumab**	60	10.7	1.06 (0.80-1.40)	1.26 (0.96-1.67)	1.28 (0.97-1.70)	1.14 (0.82-1.58)
**Abatacept**	125	16.1	1.61 (1.30-2.00)	1.08 (0.86-1.36)	1.08 (0.86-1.36)	1.14 (0.87-1.48)
**Rituximab**	290	21.0	2.07 (1.75-2.46)	1.23 (1.03-1.48)	1.24 (1.04-1.49)	1.27 (1.04-1.55)
**Tocilizumab**	84	11.4	1.13 (0.88-1.45)	0.92 (0.71-1.20)	0.93 (0.72-1.21)	0.97 (0.70-1.33)
**Sarilumab**	4	13.1				
**Baricitinib**	30	10.7	1.10 (0.75-1.61)	0.81 (0.55-1.20)	0.82 (0.54-1.22)	0.83 (0.49-1.42)
**Tofacitinib**	7	12.9	1.32 (0.62-2.81)	1.02 (0.49-2.16)	1.05 (0.50-2.21)	0.78 (0.31-1.99)
**Acute Coronary Syndrome**						
**Etanercept**	127	5.2	1.0 (Ref.)			
**Adalimumab**	74	6.1	1.16 (0.87-1.54)	1.17 (0.87-1.56)	1.15 (0.86-1.55)	1.00 (0.73-1.38)
**Infliximab**	44	5.7	1.08 (0.77-1.52)	0.99 (0.70-1.41)	1.00 (0.70-1.41)	0.96 (0.61-1.52)
**Certolizumab pegol**	37	6.3	1.20 (0.83-1.73)	1.29 (0.89-1.87)	1.29 (0.88-1.88)	1.20 (0.81-1.80)
**Golimumab**	31	5.4	1.04 (0.70-1.53)	1.21 (0.82-1.79)	1.23 (0.83-1.83)	1.01 (0.64-1.59)
**Abatacept**	65	8.2	1.55 (1.15-2.10)	1.11 (0.80-1.52)	1.11 (0.81-1.53)	1.10 (0.76-1.59)
**Rituximab**	160	11.2	2.14 (1.70-2.71)	1.40 (1.09-1.80)	1.41 (1.10-1.81)	1.31 (1.00-1.71)
**Tocilizumab**	39	5.2	0.99 (0.69-1.42)	0.82 (0.56-1.20)	0.83 (0.57-1.22)	0.89 (0.56-1.41)
**Sarilumab**	1	3.3				
**Baricitinib**	12	4.2	0.79 (0.44-1.43)	0.62 (0.34-1.13)	0.62 (0.33-1.14)	0.42 (0.21-0.83)
**Tofacitinib**	4	7.3				
**Stroke**						
**Etanercept**	98	4.0	1.0 (Ref.)			
**Adalimumab**	61	5.0	1.25 (0.91-1.72)	1.25 (0.91-1.73)	1.24 (0.90-1.72)	1.21 (0.84-1.74)
**Infliximab**	41	5.3	1.32 (0.91-1.90)	1.22 (0.84-1.77)	1.24 (0.85-1.80)	1.25 (0.77-2.02)
**Certolizumab pegol**	33	5.6	1.41 (0.95-2.08)	1.48 (1.01-2.18)	1.49 (1.01-2.20)	1.38 (0.90-2.10)
**Golimumab**	29	5.0	1.25 (0.83-1.90)	1.46 (0.96-2.20)	1.49 (0.98-2.27)	1.59 (0.97-2.61)
**Abatacept**	52	6.4	1.62 (1.16-2.26)	1.07 (0.75-1.52)	1.07 (0.75-1.53)	1.15 (0.76-1.74)
**Rituximab**	118	8.1	2.00 (1.53-2.62)	1.18 (0.89-1.56)	1.19 (0.89-1.58)	1.28 (0.95-1.74)
**Tocilizumab**	40	5.3	1.31 (0.91-1.89)	1.07 (0.73-1.57)	1.09 (0.74-1.60)	1.08 (0.67-1.75)
**Sarilumab**	3	9.5				
**Baricitinib**	13	4.5	1.20 (0.67-2.14)	0.83 (0.46-1.52)	0.81 (0.44-1.51)	0.91 (0.42-1.96)
**Tofacitinib**	1	1.8				

**Fatal cardiovascular event**						
**Etanercept**	68	2.7	1.0 (Ref.)			
**Adalimumab**	36	2.9	1.08 (0.72-1.63)	1.18 (0.79-1.77)	1.13 (0.75-1.70)	0.85 (0.53-1.37)
**Infliximab**	25	3.1	1.15 (0.73-1.82)	1.02 (0.65-1.61)	0.97 (0.61-1.55)	0.79 (0.42-1.50)
**Certolizumab pegol**	18	3.0	1.08 (0.65-1.82)	1.17 (0.70-1.96)	1.12 (0.66-1.90)	0.82 (0.47-1.43)
**Golimumab**	17	2.9	1.04 (0.61-1.77)	1.36 (0.79-2.32)	1.32 (0.77-2.28)	0.94 (0.51-1.76)
**Abatacept**	33	4.0	1.48 (0.98-2.24)	0.80 (0.52-1.25)	0.81 (0.52-1.28)	0.72 (0.44-1.18)
**Rituximab**	96	6.4	2.26 (1.65-3.09)	1.05 (0.75-1.47)	1.02 (0.73-1.44)	0.94 (0.65-1.36)
**Tocilizumab**	41	5.3	1.91 (1.30-2.82)	1.53 (1.00-2.35)	1.51 (0.98-2.32)	1.55 (0.96-2.50)
**Sarilumab**	0	0.0				
**Baricitinib**	11	3.8	1.58 (0.83-3.01)	1.02 (0.52-1.99)	1.15 (0.57-2.33)	1.07 (0.42-2.73)
**Tofacitinib**	2	3.5				
**Liver disease**						
**Etanercept**	29	1.2	1.0 (Ref.)			
**Adalimumab**	14	1.1	0.95 (0.50-1.80)	0.94 (0.49-1.79)	0.91 (0.47-1.77)	0.73 (0.36-1.47)
**Infliximab**	18	2.3	1.92 (1.07-3.46)	1.98 (1.08-3.64)	2.05 (1.11-3.79)	1.87 (0.86-4.04)
**Certolizumab pegol**	14	2.4	1.97 (1.04-3.73)	2.18 (1.15-4.15)	2.36 (1.23-4.52)	1.76 (0.87-3.54)
**Golimumab**	3	0.5				
**Abatacept**	14	1.7	1.43 (0.75-2.72)	1.22 (0.61-2.44)	1.23 (0.62-2.47)	1.60 (0.72-3.57)
**Rituximab**	33	2.2	1.90 (1.16-3.12)	1.55 (0.89-2.68)	1.57 (0.91-2.72)	1.58 (0.88-2.85)
**Tocilizumab**	11	1.4	1.21 (0.60-2.42)	1.12 (0.53-2.37)	1.15 (0.54-2.42)	1.06 (0.43-2.63)
**Sarilumab**	2	6.4				
**Baricitinib**	5	1.7	1.41 (0.54-3.69)	1.20 (0.45-3.20)	0.99 (0.35-2.82)	0.62 (0.18-2.16)
**Tofacitinib**	1	1.8				
**All-cause mortality**						
**Etanercept**	219	8.8	1.0 (Ref.)			
**Adalimumab**	100	8.0	0.93 (0.73-1.18)	1.03 (0.81-1.30)	1.02 (0.81-1.29)	0.88 (0.67-1.16)
**Infliximab**	92	11.6	1.31 (1.03-1.67)	1.14 (0.89-1.46)	1.16 (0.91-1.49)	1.05 (0.75-1.47)
**Certolizumab pegol**	55	9.2	1.03 (0.77-1.38)	1.12 (0.84-1.49)	1.12 (0.84-1.50)	0.98 (0.71-1.35)
**Golimumab**	50	8.6	0.95 (0.70-1.29)	1.19 (0.87-1.61)	1.23 (0.90-1.67)	1.08 (0.75-1.55)
**Abatacept**	140	16.8	1.99 (1.61-2.45)	1.27 (1.01-1.59)	1.27 (1.01-1.60)	1.32 (1.01-1.73)
**Rituximab**	366	24.3	2.65 (2.24-3.14)	1.36 (1.14-1.63)	1.38 (1.15-1.64)	1.40 (1.15-1.71)
**Tocilizumab**	112	14.5	1.62 (1.29-2.04)	1.44 (1.13-1.84)	1.47 (1.15-1.88)	1.41 (1.06-1.88)
**Sarilumab**	3	9.5				
**Baricitinib**	55	18.8	2.60 (1.93-3.51)	1.90 (1.38-2.61)	1.86 (1.33-2.60)	2.27 (1.51-3.40)
**Tofacitinib**	5	8.7	1.23 (0.51-2.98)	1.02 (0.43-2.41)	1.04 (0.44-2.45)	1.09 (0.40-2.92)
**Any hospitalization**						
**Etanercept**	2465	128.2	1.0 (Ref.)			
**Adalimumab**	1329	139.3	1.04 (0.97-1.11)	1.07 (1.00-1.15)	1.07 (1.00-1.14)	1.04 (0.97-1.12)
**Infliximab**	961	164.6	1.27 (1.18-1.36)	1.26 (1.17-1.36)	1.21 (1.12-1.31)	1.18 (1.07-1.29)
**Certolizumab pegol**	679	158.0	1.24 (1.14-1.35)	1.26 (1.16-1.38)	1.20 (1.10-1.31)	1.15 (1.05-1.26)
**Golimumab**	570	128.3	1.04 (0.95-1.14)	1.10 (1.01-1.21)	1.06 (0.96-1.16)	1.06 (0.96-1.17)
**Abatacept**	1183	202.7	1.50 (1.40-1.61)	1.08 (1.01-1.16)	1.08 (1.01-1.16)	1.08 (0.99-1.18)
**Rituximab**	2091	232.3	1.82 (1.72-1.93)	1.25 (1.18-1.34)	1.23 (1.15-1.31)	1.28 (1.19-1.37)
**Tocilizumab**	973	182.2	1.41 (1.31-1.52)	1.12 (1.04-1.22)	1.11 (1.02-1.20)	1.05 (0.96-1.16)
**Sarilumab**	53	194.1	1.19 (0.91-1.55)	0.92 (0.69-1.23)	1.05 (0.78-1.41)	1.40 (0.98-2.00)
**Baricitinib**	438	179.6	1.19 (1.07-1.31)	0.90 (0.80-1.00)	1.00 (0.90-1.12)	0.91 (0.80-1.04)
**Tofacitinib**	94	198.4	1.29 (1.05-1.59)	0.96 (0.77-1.19)	1.07 (0.86-1.33)	0.69 (0.49-0.96)
**Serious infection**						
**Etanercept**	571	24.8	1.0 (Ref.)			
**Adalimumab**	343	29.6	1.17 (1.02-1.33)	1.20 (1.05-1.37)	1.21 (1.06-1.39)	1.12 (0.96-1.31)
**Infliximab**	265	36.0	1.45 (1.26-1.68)	1.41 (1.21-1.63)	1.37 (1.18-1.59)	1.32 (1.09-1.60)
**Certolizumab pegol**	167	30.4	1.25 (1.05-1.48)	1.28 (1.07-1.53)	1.22 (1.02-1.46)	1.10 (0.91-1.33)
**Golimumab**	134	24.9	1.03 (0.85-1.24)	1.13 (0.93-1.37)	1.09 (0.90-1.32)	1.07 (0.86-1.32)
**Abatacept**	278	38.6	1.53 (1.32-1.76)	1.12 (0.97-1.30)	1.11 (0.96-1.29)	1.08 (0.91-1.29)
**Rituximab**	609	47.5	1.96 (1.75-2.19)	1.29 (1.14-1.46)	1.28 (1.13-1.44)	1.31 (1.15-1.50)
**Tocilizumab**	235	33.5	1.36 (1.17-1.58)	1.09 (0.93-1.27)	1.07 (0.91-1.26)	0.98 (0.80-1.19)
**Sarilumab**	7	23.3	0.80 (0.38-1.67)	0.60 (0.28-1.27)	0.72 (0.34-1.53)	0.61 (0.24-1.53)
**Baricitinib**	105	39.5	1.43 (1.16-1.77)	1.11 (0.89-1.38)	1.26 (1.00-1.58)	0.98 (0.75-1.29)
**Tofacitinib**	25	47.3	1.69 (1.14-2.52)	1.30 (0.88-1.94)	1.46 (0.98-2.18)	0.89 (0.47-1.69)
**Tuberculosis**						

**Etanercept**	1	0.0				
**Adalimumab**	3	0.2				
**Infliximab**	4	0.5				
**Certolizumab pegol**	4	0.7				
**Golimumab**	2	0.3				
**Abatacept**	0	0.0				
**Rituximab**	2	0.1				
**Tocilizumab**	0	0.0				
**Sarilumab**	0	0.0				
**Baricitinib**	0	0.0				
**Tofacitinib**	0	0.0				
**Herpes zoster**						
**Etanercept**	50	2.0	1.0 (Ref.)			
**Adalimumab**	38	3.1	1.50 (0.99-2.30)	1.53 (1.01-2.34)	1.48 (0.97-2.27)	1.48 (0.90-2.43)
**Infliximab**	27	3.4	1.68 (1.06-2.69)	1.67 (1.03-2.69)	1.62 (1.00-2.64)	1.64 (0.90-2.99)
**Certolizumab pegol**	18	3.0	1.50 (0.87-2.57)	1.55 (0.90-2.66)	1.49 (0.86-2.58)	1.32 (0.73-2.38)
**Golimumab**	15	2.6	1.28 (0.72-2.28)	1.36 (0.76-2.45)	1.34 (0.74-2.41)	1.20 (0.63-2.28)
**Abatacept**	23	2.8	1.39 (0.85-2.28)	0.98 (0.59-1.63)	0.99 (0.59-1.64)	0.89 (0.49-1.64)
**Rituximab**	55	3.7	1.82 (1.24-2.68)	1.21 (0.80-1.82)	1.21 (0.80-1.82)	1.21 (0.77-1.91)
**Tocilizumab**	14	1.8	0.90 (0.49-1.62)	0.71 (0.38-1.33)	0.70 (0.37-1.31)	1.06 (0.52-2.17)
**Sarilumab**	1	3.2				
**Baricitinib**	29	10.0	5.01 (3.15-7.96)	3.73 (2.27-6.13)	4.13 (2.37-7.19)	3.82 (2.05-7.09)
**Tofacitinib**	8	14.1	7.04 (3.33-14.90)	5.28 (2.39-11.67)	5.92 (2.64-13.30)	4.00 (1.59-10.06)
**Depression**						
**Etanercept**	91	3.8	1.0 (Ref.)			
**Adalimumab**	42	3.5	0.92 (0.64-1.32)	0.95 (0.66-1.37)	0.94 (0.65-1.37)	0.97 (0.65-1.45)
**Infliximab**	31	4.0	1.08 (0.72-1.62)	1.07 (0.71-1.62)	1.04 (0.69-1.58)	1.12 (0.65-1.93)
**Certolizumab pegol**	17	3.0	0.81 (0.48-1.35)	0.75 (0.45-1.26)	0.73 (0.43-1.23)	0.64 (0.37-1.11)
**Golimumab**	13	2.3	0.63 (0.35-1.13)	0.63 (0.35-1.13)	0.63 (0.35-1.13)	0.59 (0.32-1.10)
**Abatacept**	40	5.0	1.29 (0.89-1.87)	1.13 (0.77-1.65)	1.13 (0.77-1.65)	0.96 (0.60-1.53)
**Rituximab**	80	5.6	1.52 (1.13-2.06)	1.32 (0.96-1.83)	1.31 (0.95-1.82)	1.37 (0.95-1.97)
**Tocilizumab**	42	5.7	1.53 (1.06-2.20)	1.35 (0.92-1.99)	1.35 (0.92-1.99)	1.28 (0.82-1.99)
**Sarilumab**	1	3.3				
**Baricitinib**	18	6.5	1.44 (0.87-2.40)	1.34 (0.79-2.28)	1.51 (0.85-2.67)	1.15 (0.63-2.08)
**Tofacitinib**	5	9.0	2.00 (0.81-4.93)	1.60 (0.64-3.96)	1.76 (0.70-4.41)	0.98 (0.30-3.17)
**Suicidality**						
**Etanercept**	35	1.4	1.0 (Ref.)			
**Adalimumab**	21	1.7	1.18 (0.69-2.01)	1.19 (0.69-2.04)	1.29 (0.74-2.22)	1.03 (0.56-1.90)
**Infliximab**	13	1.7	1.15 (0.61-2.17)	1.07 (0.56-2.06)	1.11 (0.57-2.14)	1.32 (0.56-3.14)
**Certolizumab pegol**	7	1.2	0.82 (0.37-1.83)	0.77 (0.34-1.72)	0.71 (0.31-1.63)	0.80 (0.33-1.97)
**Golimumab**	6	1.0	0.72 (0.30-1.71)	0.71 (0.30-1.69)	0.68 (0.28-1.62)	0.57 (0.22-1.48)
**Abatacept**	13	1.6	1.10 (0.59-2.09)	0.94 (0.48-1.84)	0.91 (0.47-1.76)	1.05 (0.45-2.46)
**Rituximab**	23	1.5	1.09 (0.64-1.84)	0.92 (0.53-1.60)	0.93 (0.53-1.63)	0.78 (0.41-1.47)
**Tocilizumab**	18	2.4	1.64 (0.93-2.91)	1.44 (0.79-2.64)	1.45 (0.80-2.64)	1.17 (0.58-2.34)
**Sarilumab**	2	6.5				
**Baricitinib**	6	2.1	1.42 (0.60-3.38)	1.38 (0.55-3.44)	1.71 (0.65-4.47)	0.94 (0.37-2.40)
**Tofacitinib**	1	1.8				

Notes: [a)] model included (i.e., adjusted for) age, sex, immigrant status, highest achieved education, RF/ACPA, RA duration, previous b/tsDMARD use, co-medication with conventional synthetic DMARDs and glucocorticosteroids, the 28-joint disease activity score (DAS28-CRP),the Health Assessment Questionnaire-Disability Index (HAQ), history of malignancy, infections, joint surgery, chronic pulmonary disease, diabetes, cardiovascular disease, depression, and the sum of days hospitalized in last five years.

Figure S1. MACE component outcomes among all Swedish RA patients who started b/tsDMARD 2010-2020.

Outcome	Events	Crude IR	wIR	wHR (95% CI)
**Acute coronary syndrome**				
Etanercept	127	5.2	6.6	1.0 (Ref.)
Adalimumab	74	6.1	6.7	1.00 (0.73-1.38)
Infliximab	44	5.7	6.4	0.96 (0.61-1.52)
Certolizumab	37	6.3	8.0	1.20 (0.81-1.80)
Golimumab	31	5.4	6.7	1.01 (0.64-1.59)
Abatacept	65	8.2	7.3	1.10 (0.76-1.59)
Rituximab	160	11.2	8.7	1.31 (1.00-1.71)
Tocilizumab	39	5.2	5.9	0.89 (0.56-1.41)
Sarilumab	1	3.3	4.3	
Baricitinib	12	4.2	2.8	0.42 (0.21-0.83)
Tofacitinib	4	7.3	4.6	
**Stroke**				
Etanercept	98	4.0	4.8	1.0 (Ref.)
Adalimumab	61	5.0	5.7	1.21 (0.84-1.74)
Infliximab	41	5.3	5.9	1.25 (0.77-2.02)
Certolizumab	33	5.6	6.5	1.38 (0.90-2.10)
Golimumab	29	5.0	7.6	1.59 (0.97-2.61)
Abatacept	52	6.4	5.4	1.15 (0.76-1.74)
Rituximab	118	8.1	6.2	1.28 (0.95-1.74)
Tocilizumab	40	5.3	5.2	1.08 (0.67-1.75)
Sarilumab	3	9.5	11.2	
Baricitinib	13	4.5	4.1	0.91 (0.42-1.96)
Tofacitinib	1	1.8	0.6	
**Fatal cardiovascular event**				
Etanercept	68	2.7	4.4	1.0 (Ref.)
Adalimumab	36	2.9	3.7	0.85 (0.53-1.37)
Infliximab	25	3.1	3.5	0.79 (0.42-1.50)
Certolizumab	18	3.0	3.7	0.82 (0.47-1.43)
Golimumab	17	2.9	4.3	0.94 (0.51-1.76)
Abatacept	33	4.0	3.1	0.72 (0.44-1.18)
Rituximab	96	6.4	4.4	0.94 (0.65-1.36)
Tocilizumab	41	5.3	7.0	1.55 (0.96-2.50)
Sarilumab	0	0.0	0.0	
Baricitinib	11	3.8	4.0	1.07 (0.42-2.73)
Tofacitinib	2	3.5	3.9	

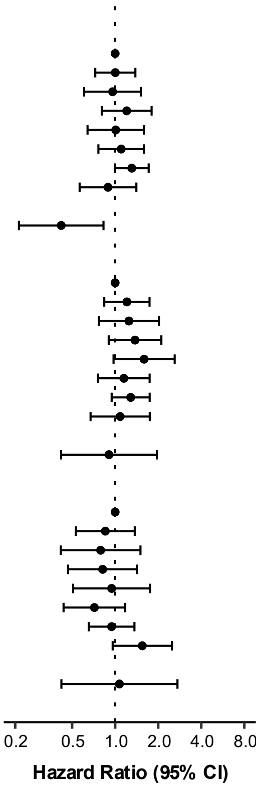

Crude and weighted incidence rate per 1000 person-years of cardiovascular outcomes by b/tsDMARD, and adjusted hazard ratios versus etanercept. wIR, inverse probability of treatment weighted incidence rate per 1000 person-years, adjusted for demographics, RA clinical characteristics, and comorbidity; wHR, weighted hazard ratio from Cox regression.

Figure S2. Incidence rate of safety outcomes comparing Swedish RA patients who started b/tsDMARD 2010-2020 to b/tsDMARD-naive patients with RA.

Outcome	Events	IR	HR (95% CI)
**Major adverse cardiovascular event**			
RA, bionaïve	8989	24.8	1.0 (Ref.)
RA, b/tsDMARD treated	1147	13.1	0.89 (0.83-0.95)
**Serious Infection**			
RA, bionaïve	12859	36.9	1.0 (Ref.)
RA, b/tsDMARD treated	2739	32.8	1.18 (1.13-1.23)
**Diagnosed herpes zoster**			
RA, bionaïve	903	2.3	1.0 (Ref.)
RA, b/tsDMARD treated	278	3.1	1.68 (1.45-1.93)
**Tuberculosis**			
RA, bionaïve	21	0.1	1.0 (Ref.)
RA, b/tsDMARD treated	16	0.2	2.37 (1.21-4.64)
**Liver disease**			
RA, bionaïve	774	1.9	1.0 (Ref.)
RA, b/tsDMARD treated	144	1.6	0.90 (0.75-1.09)
**Diagnosed depression**			
RA, bionaïve	1793	4.6	1.0 (Ref.)
RA, b/tsDMARD treated	380	4.3	0.81 (0.73-0.91)
**Suicide (incl. attempted)**			
RA, bionaïve	673	1.7	1.0 (Ref.)
RA, b/tsDMARD treated	145	1.6	0.90 (0.74-1.08)
**Any hospitalization**			
RA, bionaïve	38575	167.7	1.0 (Ref.)
RA, b/tsDMARD treated	10836	162.4	1.04 (1.01-1.06)
**All-cause mortality**			
RA, bionaïve	16036	39.7	1.0 (Ref.)
RA, b/tsDMARD treated	1197	13.0	0.65 (0.61-0.69)

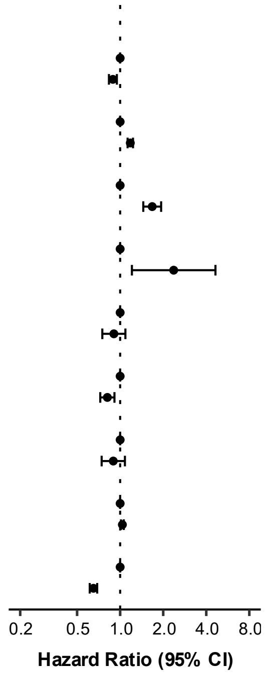

IR: age and sex-standardized incidence rate per 1000 person-years, and age-sex adjusted hazard ratios from Cox regression.

BMJ Publishing Group Limited (BMJ) disclaims all liability and responsibility arising from any reliance placed on this supplemental material which has been supplied by the author(s)

Figure S3. Crude and weighted incidence rate per 1000 person-years of selected safety outcomes by b/tsDMARD, and adjusted hazard ratios versus etanercept, among all Swedish RA patients who started treatment *2017-2020*, from JAKi market entry, followed until 30 June 2021.

Outcome	Events	Crude IR	wIR	wHR (95% CI)
**Stop for adverse events**				
Etanercept	109	14.7	26.1	
Adalimumab	129	35.6	41.5	1.33 (1.00-1.77)
Infliximab	29	28.5	75.1	2.63 (1.70-4.06)
Certolizumab	33	69.4	97.9	3.38 (2.23-5.11)
Golimumab	18	89.3	115.0	4.10 (2.34-7.19)
Abatacept	114	57.1	37.2	1.36 (1.02-1.81)
Rituximab	57	23.0	18.9	0.72 (0.50-1.04)
Tocilizumab	55	46.9	29.9	1.13 (0.78-1.63)
Sarilumab	30	94.7	57.4	1.77 (1.08-2.90)
Baricitinib	114	39.0	23.0	0.82 (0.60-1.11)
Tofacitinib	52	95.9	50.2	1.77 (1.15-2.74)
**Major adverse cardiovascular event**				
Etanercept	68	9.5	12.5	1.0 (Ref.)
Adalimumab	36	10.3	11.0	0.92 (0.58-1.46)
Infliximab	12	12.1	8.9	0.73 (0.38-1.42)
Certolizumab	4	8.5	10.5	
Golimumab	2	10.5	11.4	
Abatacept	40	21.4	19.2	1.55 (0.96-2.52)
Rituximab	42	18.0	13.4	1.06 (0.69-1.65)
Tocilizumab	14	12.5	11.7	0.93 (0.47-1.85)
Sarilumab	4	13.1	15.8	
Baricitinib	30	10.7	9.9	0.80 (0.45-1.42)
Tofacitinib	6	11.7	7.7	0.63 (0.21-1.84)
**Serious Infection**				
Etanercept	159	22.7	28.1	1.0 (Ref.)
Adalimumab	93	26.6	30.4	1.01 (0.75-1.37)
Infliximab	42	44.1	43.9	1.51 (0.98-2.35)
Certolizumab	10	21.5	28.8	0.98 (0.50-1.95)
Golimumab	4	21.4	32.6	
Abatacept	65	37.4	33.5	1.17 (0.83-1.65)
Rituximab	102	46.0	39.3	1.40 (1.04-1.87)
Tocilizumab	44	40.2	37.7	1.33 (0.85-2.09)
Sarilumab	7	23.3	21.8	0.71 (0.28-1.83)
Baricitinib	105	39.5	33.6	1.16 (0.85-1.58)
Tofacitinib	24	48.0	29.9	1.03 (0.52-2.04)
**Diagnosed herpes zoster**				
Etanercept	15	2.0	2.9	1.0 (Ref.)
Adalimumab	8	2.2	2.4	0.82 (0.31-2.15)
Infliximab	8	8.0	10.3	3.56 (1.16-10.96)
Certolizumab	0	0.0	0.0	
Golimumab	0	0.0	0.0	
Abatacept	4	2.0	2.1	
Rituximab	8	3.3	2.2	0.76 (0.29-2.01)
Tocilizumab	2	1.7	3.8	
Sarilumab	1	3.2	0.9	
Baricitinib	29	10.0	9.8	3.20 (1.42-7.21)
Tofacitinib	8	14.9	10.7	3.56 (1.24-10.28)

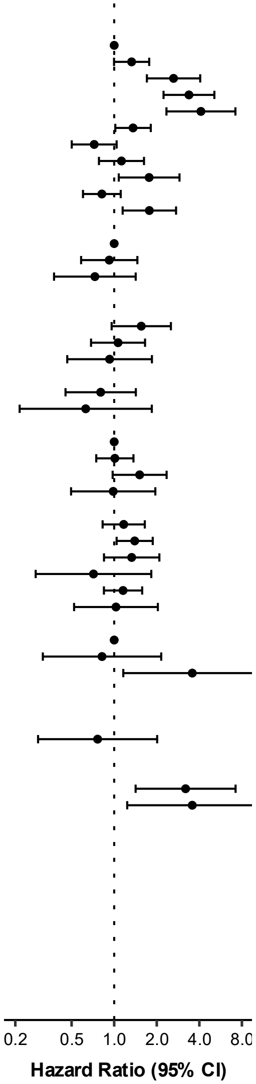

Outcome	Events	Crude IR	wIR	wHR (95% CI)
**Liver disease**				
Etanercept	13	1.8	2.8	1.0 (Ref.)
Adalimumab	4	1.1	1.0	
Infliximab	2	2.0	3.0	
Certolizumab	2	4.2	6.2	
Golimumab	0	0.0	0.0	
Abatacept	6	3.1	3.7	1.31 (0.39-4.40)
Rituximab	5	2.1	1.6	0.58 (0.19-1.81)
Tocilizumab	2	1.7	3.9	
Sarilumab	2	6.4	5.7	
Baricitinib	5	1.7	1.0	0.34 (0.09-1.29)
Tofacitinib	1	1.9	0.7	
**Diagnosed depression**				
Etanercept	27	3.8	4.5	1.0 (Ref.)
Adalimumab	11	3.2	3.3	0.63 (0.29-1.36)
Infliximab	2	2.0	2.1	
Certolizumab	3	6.7	5.4	
Golimumab	0	0.0	0.0	
Abatacept	4	2.1	2.4	
Rituximab	11	4.7	4.9	1.07 (0.41-2.77)
Tocilizumab	9	8.1	7.9	1.73 (0.69-4.35)
Sarilumab	1	3.3	1.3	
Baricitinib	18	6.5	5.3	1.08 (0.52-2.25)
Tofacitinib	5	9.5	4.8	0.98 (0.28-3.41)
**Suicide (incl. attempted)**				
Etanercept	10	1.4	1.5	1.0 (Ref.)
Adalimumab	5	1.4	1.4	0.94 (0.26-3.32)
Infliximab	1	1.0	3.2	
Certolizumab	1	2.1	2.1	
Golimumab	0	0.0	0.0	
Abatacept	3	1.5	4.1	
Rituximab	3	1.2	0.9	
Tocilizumab	0	0.0	0.0	
Sarilumab	2	6.5	2.8	
Baricitinib	6	2.1	1.5	1.06 (0.31-3.69)
Tofacitinib	1	1.9	0.3	
**Any hospitalization**				
Etanercept	788	122.9	146.1	1.0 (Ref.)
Adalimumab	449	138.7	168.1	1.07 (0.94-1.21)
Infliximab	134	156.7	156.4	1.03 (0.81-1.31)
Certolizumab	85	216.1	213.2	1.39 (1.09-1.77)
Golimumab	26	152.9	224.8	1.49 (0.98-2.25)
Abatacept	316	196.9	164.4	1.10 (0.94-1.30)
Rituximab	416	221.4	183.5	1.25 (1.08-1.44)
Tocilizumab	179	186.5	180.5	1.22 (0.98-1.52)
Sarilumab	53	194.1	274.5	1.71 (1.19-2.46)
Baricitinib	438	179.6	164.2	1.09 (0.93-1.26)
Tofacitinib	87	192.8	119.2	0.79 (0.56-1.13)
**All-cause mortality**				
Etanercept	52	7.0	9.9	1.0 (Ref.)
Adalimumab	19	5.2	7.1	0.90 (0.48-1.70)
Infliximab	14	13.8	12.4	1.36 (0.61-3.01)
Certolizumab	5	10.5	15.0	1.72 (0.65-4.52)
Golimumab	0	0.0	0.0	
Abatacept	41	20.5	20.3	2.15 (1.30-3.56)
Rituximab	50	20.2	16.5	1.67 (1.05-2.63)
Tocilizumab	17	14.5	16.7	1.70 (0.87-3.32)
Sarilumab	3	9.5	9.8	
Baricitinib	55	18.8	22.0	2.43 (1.48-4.00)
Tofacitinib	4	7.4	9.1	

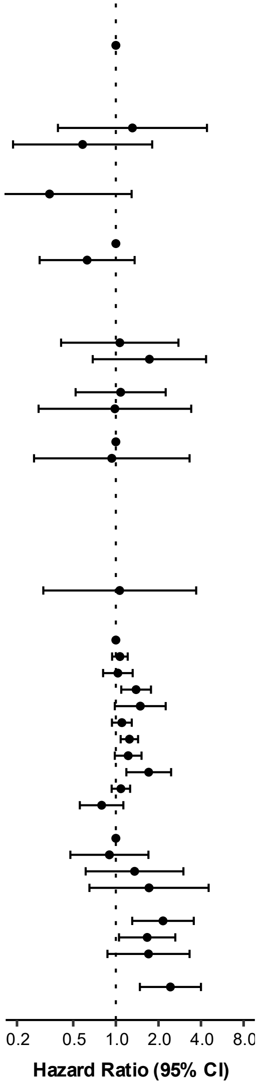

BMJ Publishing Group Limited (BMJ) disclaims all liability and responsibility arising from any reliance placed on this supplemental material which has been supplied by the author(s)

Figure S4. Crude and weighted incidence rate per 1000 person-years of selected safety outcomes by b/tsDMARD, and adjusted hazard ratios versus etanercept, among all Swedish RA patients who started treatment 2010-2020, followed *until the COVID-19 pandemic, 28 Feb 2020.*

Outcome	Events	Crude IR	wIR	wHR (95% CI)
**Stop for adverse events**				
Etanercept	397	19.9	32.9	
Adalimumab	291	30.9	38.0	1.16 (0.98-1.37)
Infliximab	92	12.9	40.6	1.20 (0.95-1.53)
Certolizumab	191	35.8	46.2	1.49 (1.24-1.79)
Golimumab	177	34.0	41.9	1.34 (1.11-1.63)
Abatacept	411	59.8	40.0	1.17 (1.01-1.36)
Rituximab	287	22.5	18.8	0.63 (0.53-0.74)
Tocilizumab	223	33.4	22.0	0.70 (0.58-0.83)
Sarilumab	13	104.6	64.1	1.16 (0.59-2.25)
Baricitinib	78	50.4	29.7	0.61 (0.45-0.81)
Tofacitinib	44	128.8	72.5	1.55 (1.01-2.38)
**Major adverse cardiovascular event**				
Etanercept	182	9.5	11.2	1.0 (Ref.)
Adalimumab	121	13.4	15.1	1.36 (1.05-1.77)
Infliximab	80	11.7	13.0	1.17 (0.83-1.66)
Certolizumab	71	13.8	16.9	1.52 (1.14-2.05)
Golimumab	53	10.5	14.1	1.27 (0.89-1.81)
Abatacept	100	15.5	14.0	1.26 (0.94-1.71)
Rituximab	256	21.8	16.3	1.45 (1.16-1.81)
Tocilizumab	72	11.3	12.6	1.13 (0.80-1.60)
Sarilumab	1	8.2	12.6	
Baricitinib	13	8.7	10.0	0.95 (0.44-2.07)
Tofacitinib	3	9.2	2.7	
**Serious Infection**				
Etanercept	488	26.3	32.4	1.0 (Ref.)
Adalimumab	280	32.0	38.9	1.20 (1.02-1.42)
Infliximab	247	37.3	42.6	1.31 (1.07-1.60)
Certolizumab	158	32.2	35.3	1.11 (0.91-1.35)
Golimumab	126	26.1	34.3	1.08 (0.86-1.34)
Abatacept	243	40.6	35.3	1.08 (0.89-1.29)
Rituximab	553	50.6	41.8	1.33 (1.15-1.53)
Tocilizumab	213	35.1	30.8	0.97 (0.78-1.19)
Sarilumab	4	33.3	43.1	
Baricitinib	76	54.2	48.4	1.28 (0.93-1.76)
Tofacitinib	19	59.5	41.3	1.11 (0.53-2.34)
**Diagnosed herpes zoster**				
Etanercept	45	2.3	2.7	1.0 (Ref.)
Adalimumab	26	2.8	3.5	1.30 (0.73-2.31)
Infliximab	24	3.4	3.7	1.35 (0.74-2.47)
Certolizumab	17	3.2	3.5	1.28 (0.70-2.36)
Golimumab	13	2.5	2.6	0.94 (0.48-1.85)
Abatacept	21	3.1	2.5	0.93 (0.49-1.75)
Rituximab	49	3.9	3.1	1.15 (0.71-1.87)
Tocilizumab	14	2.1	3.1	1.13 (0.55-2.32)
Sarilumab	1	8.1	2.2	
Baricitinib	20	13.0	12.9	4.87 (2.41-9.83)
Tofacitinib	4	11.8	10.2	

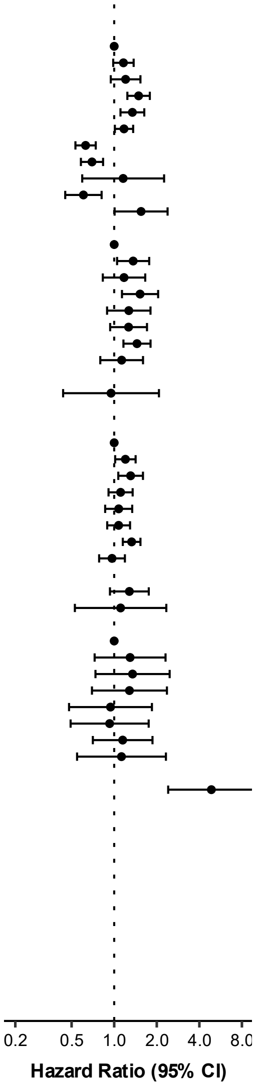

Outcome	Events	Crude IR	wIR	wHR (95% CI)
**Liver disease**				
Etanercept	22	1.1	1.4	1.0 (Ref.)
Adalimumab	11	1.2	1.2	0.83 (0.37-1.83)
Infliximab	17	2.4	3.1	2.17 (0.95-4.95)
Certolizumab	12	2.3	2.4	1.74 (0.80-3.76)
Golimumab	3	0.6	0.6	
Abatacept	7	1.0	1.1	0.74 (0.27-2.06)
Rituximab	27	2.2	2.2	1.61 (0.83-3.12)
Tocilizumab	10	1.5	1.7	1.24 (0.46-3.31)
Sarilumab	1	8.2	10.8	
Baricitinib	3	2.0	1.4	
Tofacitinib	0	0.0	0.0	
**Diagnosed depression**				
Etanercept	78	4.1	4.1	1.0 (Ref.)
Adalimumab	35	3.9	4.4	1.07 (0.69-1.67)
Infliximab	31	4.5	5.1	1.23 (0.71-2.13)
Certolizumab	15	2.9	2.5	0.62 (0.34-1.12)
Golimumab	13	2.6	2.6	0.63 (0.34-1.18)
Abatacept	36	5.5	4.4	1.06 (0.65-1.74)
Rituximab	74	6.1	5.7	1.43 (0.97-2.11)
Tocilizumab	39	6.1	5.5	1.35 (0.85-2.16)
Sarilumab	0	0.0	0.0	
Baricitinib	8	5.4	3.9	0.81 (0.36-1.86)
Tofacitinib	4	12.0	4.5	
**Suicide (incl. attempted)**				
Etanercept	26	1.3	1.4	1.0 (Ref.)
Adalimumab	16	1.7	1.8	1.21 (0.60-2.42)
Infliximab	11	1.6	1.9	1.27 (0.49-3.28)
Certolizumab	6	1.1	1.4	0.96 (0.36-2.54)
Golimumab	5	1.0	1.0	0.68 (0.24-1.93)
Abatacept	13	1.9	2.2	1.51 (0.63-3.63)
Rituximab	20	1.6	1.2	0.84 (0.42-1.66)
Tocilizumab	17	2.6	2.2	1.57 (0.75-3.29)
Sarilumab	0	0.0	0.0	
Baricitinib	5	3.3	2.7	1.68 (0.60-4.68)
Tofacitinib	0	0.0	0.0	
**Any hospitalization**				
Etanercept	2082	132.6	159.7	1.0 (Ref.)
Adalimumab	1075	150.2	181.1	1.13 (1.04-1.22)
Infliximab	910	170.7	196.6	1.21 (1.09-1.33)
Certolizumab	638	162.8	180.3	1.16 (1.05-1.27)
Golimumab	542	133.6	162.7	1.06 (0.96-1.17)
Abatacept	1049	213.0	178.5	1.09 (1.00-1.20)
Rituximab	1906	244.1	199.3	1.30 (1.21-1.40)
Tocilizumab	912	195.1	169.2	1.08 (0.98-1.20)
Sarilumab	26	227.1	313.8	1.45 (0.86-2.44)
Baricitinib	300	222.3	203.9	1.02 (0.87-1.20)
Tofacitinib	66	225.5	142.1	0.73 (0.48-1.09)
**All-cause mortality**				
Etanercept	165	8.3	11.3	1.0 (Ref.)
Adalimumab	76	8.1	10.9	0.96 (0.70-1.32)
Infliximab	76	10.7	12.3	1.10 (0.76-1.59)
Certolizumab	46	8.6	11.4	0.98 (0.69-1.40)
Golimumab	43	8.2	12.9	1.10 (0.74-1.64)
Abatacept	101	14.7	14.3	1.29 (0.95-1.76)
Rituximab	275	21.6	14.9	1.25 (0.99-1.57)
Tocilizumab	94	14.1	17.3	1.50 (1.10-2.04)
Sarilumab	1	8.0	12.3	
Baricitinib	22	14.2	20.6	2.55 (1.40-4.64)
Tofacitinib	2	5.9	6.0	

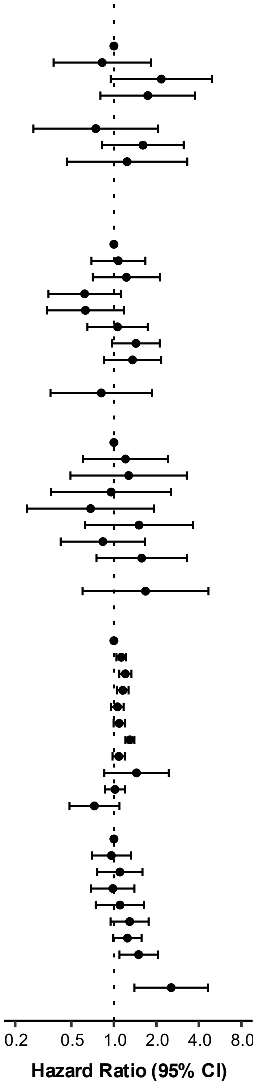

Supplement: Supplementary data [file ard-2022-223762supp001.pdf]
